# Transcriptomic Analyses of Sexual Dimorphism of the Zebrafish Liver and the Effect of Sex Hormones

**DOI:** 10.1371/journal.pone.0053562

**Published:** 2013-01-17

**Authors:** Weiling Zheng, Hongyan Xu, Siew Hong Lam, Huaien Luo, R. Krishna Murthy Karuturi, Zhiyuan Gong

**Affiliations:** 1 Department of Biological Sciences, National University of Singapore, Singapore, Singapore; 2 Computational and Systems Biology, Genome Institute of Singapore, Singapore, Singapore; Auburn University, United States of America

## Abstract

The liver is one of the most sex-dimorphic organs in both oviparous and viviparous animals. In order to understand the molecular basis of the difference between male and female livers, high-throughput RNA-SAGE (serial analysis of gene expression) sequencing was performed for zebrafish livers of both sexes and their transcriptomes were compared. Both sexes had abundantly expressed genes involved in translation, coagulation and lipid metabolism, consistent with the general function of the liver. For sex-biased transcripts, from in addition to the high enrichment of vitellogenin transcripts in spawning female livers, which constituted nearly 80% of total mRNA, it is apparent that the female-biased genes were mostly involved in ribosome/translation, estrogen pathway, lipid transport, etc, while the male-biased genes were enriched for oxidation reduction, carbohydrate metabolism, coagulation, protein transport and localization, etc. Sexual dimorphism on xenobiotic metabolism and anti-oxidation was also noted and it is likely that retinol x receptor (RXR) and liver x receptor (LXR) play central roles in regulating the sexual differences of lipid and cholesterol metabolisms. Consistent with high ribosomal/translational activities in the female liver, female-biased genes were significantly regulated by two important transcription factors, Myc and Mycn. In contrast, Male livers showed activation of transcription factors Ppargc1b, Hnf4a, and Stat4, which regulate lipid and glucose metabolisms and various cellular activities. The transcriptomic responses to sex hormones, 17β-estradiol (E2) or 11-keto testosterone (KT11), were also investigated in both male and female livers and we found that female livers were relatively insensitive to sex hormone disturbance, while the male livers were readily affected. E2 feminized male liver by up-regulating female-biased transcripts and down-regulating male-biased transcripts. The information obtained in this study provides comprehensive insights into the sexual dimorphism of zebrafish liver transcriptome and will facilitate further development of the zebrafish as a human liver disease model.

## Introduction

The liver plays a critical role in the coordination of various physiological processes including digestion, metabolism, detoxification, biosynthesis of serum proteins, endocrine and immune response, etc. Because of the different metabolic needs for male and female reproduction, the liver is one of the most sexually dimorphic organs in terms of gene expression [1]. This is particularly prominent in oviparous species as the female liver is the main organ for production of yolk protein precursors (vitellogenins) and some zona pellucida proteins.

Recently, the zebrafish has emerged as models for liver diseases such as steatosis [2], alcoholic liver disease [3], polycystic liver disease [4], and tumorigenesis [5], [6], [7], [8], [9], [10], [11] as well as liver regeneration [12], [13] and environmental hepatotoxicity [14], [15]. Sex differences in the zebrafish transcriptome have been studied previously with the whole organism [16], [17], gonads [17], [18], [19] or other organs [20], [21]. Sexual dimorphism of gene expression in the liver has also been investigated in other fish species, including tilapia [22] and turbot [23]. Sexual dimorphism of hepatic response to dietary carbohydrate manipulation [24], brominated flame retardants [25] and perfluorononanoic acid (PFNA) [26] has also been reported in the zebrafish. One microarray-based study in zebrafish has indicated that female livers have higher levels of transcripts associated with translation, while the male up-regulated genes are associated with oxidative metabolism, carbohydrate metabolism, energy production, and amelioration of oxidative stress [24].

The available evidence indicates that sexual dimorphism in the liver is mediated via the sex hormones in both oviparous and viviparous animals [27]. In the present study, we intend to compare the transcriptomic difference between female and male livers in zebrafish using the deep sequencing technology. Our comparative transcriptomic analyses indicated functional differences in translation, carbohydrate metabolism, lipid and cholesterol metabolism, and xenobiotic metabolism between female and male zebrafish livers. Different gene expression regulatory networks for causing these differences were also identified. Furthermore, we also used female and male sex hormones to treat both male and female zebrafish and found that male liver transcriptome was readily responsive to both female and male hormones while female livers were relatively resistant to the sex hormone perturbation. Thus, our transcriptomic data presented here should provide a molecular basis for a better understanding of the sexual dimorphism of zebrafish and facilitate proper experimental design in future studies.

## Materials and Methods

### Ethics Statement

All experimental protocols were approved by Institutional Animal Care and Use Committee (IACUC) of National University of Singapore (Protocol 079/07).

### Zebrafish Treatment and Sample Collection

Four-month-old adult zebrafish were purchased from a local fish farm (Mainland Tropical Fish Farm, Singapore) and were acclimated for one week prior to experimental treatments. Fish were maintained based on the standard methods [28] and water quality was monitored daily in the zebrafish aquarium of Department of Biological Sciences, National University of Singapore. For experimental treatment, male and female fish were kept separately at room temperature (28±0.5°C) under 14 h of light and 10 h of dark cycle. 15 fish were placed in a 3-liter tank and were exposed for 48 hours in a static condition with 5 µg/L 17β-estradiol (E2) or 5 µg/L 11-keto testosterone (KT11) (Sigma-Aldrich); the relatively high concentrations of sex hormones used in the experiment was to ensure the response of the fish in the short, acute treatment. Water was changed daily with fresh sex hormone added. The same treatment was also conducted with 0.01% (v/v) DMSO as a vehicle control. A previous report indicated that 0.01% DMSO did not cause any developmental defect or induce stress protein expression in zebrafish embryos and it is within the range of recommended concentration for solvent controls in zebrafish experiments [29] There was no feeding and aeration during the short 48-hour of acute chemical exposure experiment and at the end of the exposure, the liver samples were collected for RNA isolation.

### RNA-SAGE (Serial Analysis of Gene Expression) Sequencing and Data Processing

Total RNA was extracted from individual zebrafish livers using TRIzol® Reagent (Invitrogen) and treated with DNase I (Invitrogen) to remove genomic DNA contamination. Male and female fish were discerned by morphological appearance and confirmed by presence of testis and ovary after dissection. To ascertain the correct sex, RNA samples were further confirmed by real-time RT-PCR with *vtg1* primers (Forward: 5′-GGATTCCAGAGATCACAATGT-3′; Reward: 5′-CAGTACAGCAGTGGTCTAAT-3′) as female livers contained extraordinarily high level of vitellogenin mRNA. For RNA-SAGE sequencing, equal amount of total RNA from seven individual fish liver were pooled within the same experimental group for construction of 3′ SAGE libraries. The construction of SAGE libraries and sequencing were performed using SOLiD^TM^ Analyzer 4 (Applied Biosystems) by Mission Biotech Co. Ltd, Taiwan according to manufacturer’s protocol (Applied Biosystems SOLiD SAGE Guide). Briefly, mRNA was purified using Dynabeads® Oligo(dT) EcoP (Invitrogen) and subjected to cDNA synthesis. Synthesized cDNA was digested by NlaIII and EcoP15I, and sequencing adapters were added to the cDNA fragments after the digestion. A total of six SAGE libraries were sequenced: F_DMSO (control female), F_E2 (E2-treated female), F_11KT (11KT-treated female), M_DMSO (control male), M_E2 (E2-treated male), and M_11KT (11KT-treated male).

### Sequence Tag Mapping and Annotation

50 nucleotides were sequenced from the SOLiD system and 27 nucleotide starting from CATG (based on the distance of EcoP15I recognition and cut sites) were used to map to the zebrafish Reference Sequence database (http://www.ncbi.nlm.nih.gov/RefSeq) with criteria of allowing maximum 2 nucleotide mismatches by taking account of sequencing errors and sequence polymorphism [30]. Uniquely mapped tag counts for each transcript were normalized to TPM (transcript per million) to facilitate comparison among different samples. In some cases, SAGE tags mapped to the same sequence ID were pooled.

### Identification of Differentially Expressed Genes

Differentially expressed genes were identified using edgeR, a Bioconductor package for differential expression analysis of digital gene expression data [31], [32], [33]. edgeR estimates the genewise dispersions by conditional maximum likelihood, conditioning on the total count for that gene. This method models tag counts as negative binomial (NB) distributed to account for overdispersion in the digital gene expression data. Then a common dispersion is estimated for all tags. Finally, an exact test similar to Fisher’s exact test is carried out to assess the differential expression for each gene. As we did not have replicates in this experiment, we followed the recommendation in the edgeR user guide based on the assumption that tag counts are not too small and a relatively small number of genes are differentially expressed. Genes with sum of tag counts in six samples less than 30 were removed and p value cut-off was set at 0.05.

### Real-time RT-PCR Validation

To validate RNA-SAGE data, zebrafish from the same experiment but not used for RNA-SAGE sequencing were used for real-time RT-PCR analyses. For each condition, equal amount of total RNA from three fish livers were pooled and three biological replicates from a total of nine fish were used. For each biological replicate, five technical replicates were performed. Coefficient variance for the five technical replicates was smaller than 0.05 for each group. *Ef1a* was used as the housekeeping gene. Real-time PCR was performed using SYBR Green I Master (Roche) on the LightCycler 480 (Roche) following the manufacture’s protocol.

### Gene Ontology Enrichment Analysis

Gene ontology enrichment analysis was performed using DAVID (The Database for Annotation, Visualization and Integrated Discovery) [34] with the total zebrafish genome information as the background and p-values representing a modified Fisher’s exact t-test. Gene Ontology Fat categories were used for this analysis and the GO Fat attempts to filter out the broadest terms so that they will not overshadow the more specific terms. The p value cut-off was 0.05.

### Ingenuity Pathway Analysis (IPA)

The sex-biased transcripts (p<0.1) were ranked by logarithm-transformed (base 10) p values. Positive values were given to female-biased transcripts, and negative values were given to male-biased transcripts. The ranked list was then analysed with IPA software (www.ingenuity.com) for pathway and transcription factors (TFs). The algorithm of IPA also incorporates other genes from the database to maximize the connectivity with the sex-biased transcripts to assemble a ‘focus gene network’. Networks are limited to 35 molecules each to keep them to a functional size. Network scores were generated based on the hypergeometric distribution and were calculated with the right-tailed Fisher’s exact test. In the networks, nodes represent biological entities (e.g. genes, proteins, and complexes) and edges represent interactions (e.g. induction, inhibition, binding, regulation, and phosphorylation) between nodes in the pathway. TF prediction by IPA was based on information compiled only from literature with experimental evidence. First, known targets of each transcription factor in the sex-biased transcript list were examined and compared with the direction of altered target gene expression, and finally a prediction for each transcription factor based on the direction of change was generated. The regulation z-score algorithm was used to make predictions. Only TFs with absolute z-score>2 and p-value<0.05 were considered as significantly enriched.

## Results and Discussion

### Overview of SAGE Sequencing Data

A total of six liver SAGE libraries were generated, including control female and male, E2-treated female and male, and KT11-treated female and male. For each library, between 11 and 20 million sequence reads were generated (**Table S1**). The read tags were mapped to the zebrafish RefSeq database with mapping efficiency from 29.0% to 43.4%. A total of 8,154 transcript entries were detected from the female liver, while 12,183 expressed genes from the male liver. In general, there were fewer transcripts detected in female livers probably due to the high portion of vitellogenin mRNA (78 %). However, in term of the number of genes more robustly expressed with at least 30 sequence tags detected in both SAGE libraries, the numbers were quite comparable between female and male samples (4,386 in female and 4,548 in male) (**Table S1**).

Among the top 50 expressed genes in the female and male livers, there were 34 genes overlapping, including three vitellogenin genes (*vtg1*, *vtg4*, *vtg5*), 11 ribosomal protein genes (*rplp0*, *rps25*, *rps15a*, *rps10*, *rps27a*, *rpl7*, *rps29*, *rpl31*, *rpl6*, *rps27.1*, *rpl10*), six complement factor genes (*fga*, *fgg*, *c3a*, *LOC100334885*, *cfb*, *plg*), four lipoprotein genes (*fabp10a*, *apobl*, *apoa2*, *apoc1l*), two glycoprotein genes (*tfa*, *ahsg*), and a few others (*il7r*, *a2m*, *tpt1*, *mibp2*, *gpx4a* and three unannotated genes). In the female liver, the top four transcripts were all vitellogenin genes, including *vtg1*, *vtg4*, *vtg5* and *vtg2*. In the top 50 expressed transcripts in the female liver, 23 of them were ribosomal protein genes. On the other hand, in the top 50 expressed transcripts in the male liver, there were only 12 ribosomal protein genes, indicating a strong translational activity in female livers. It is also interesting to note that three vitellogenin transcripts (*vtg1, vtg4, vtg5*) also appeared in the top 50 list, implying potentially yolk-unrelated functions of vitellogenins, such as lipid transportation [35] and anti-microbes [36].

**Figure 1:**
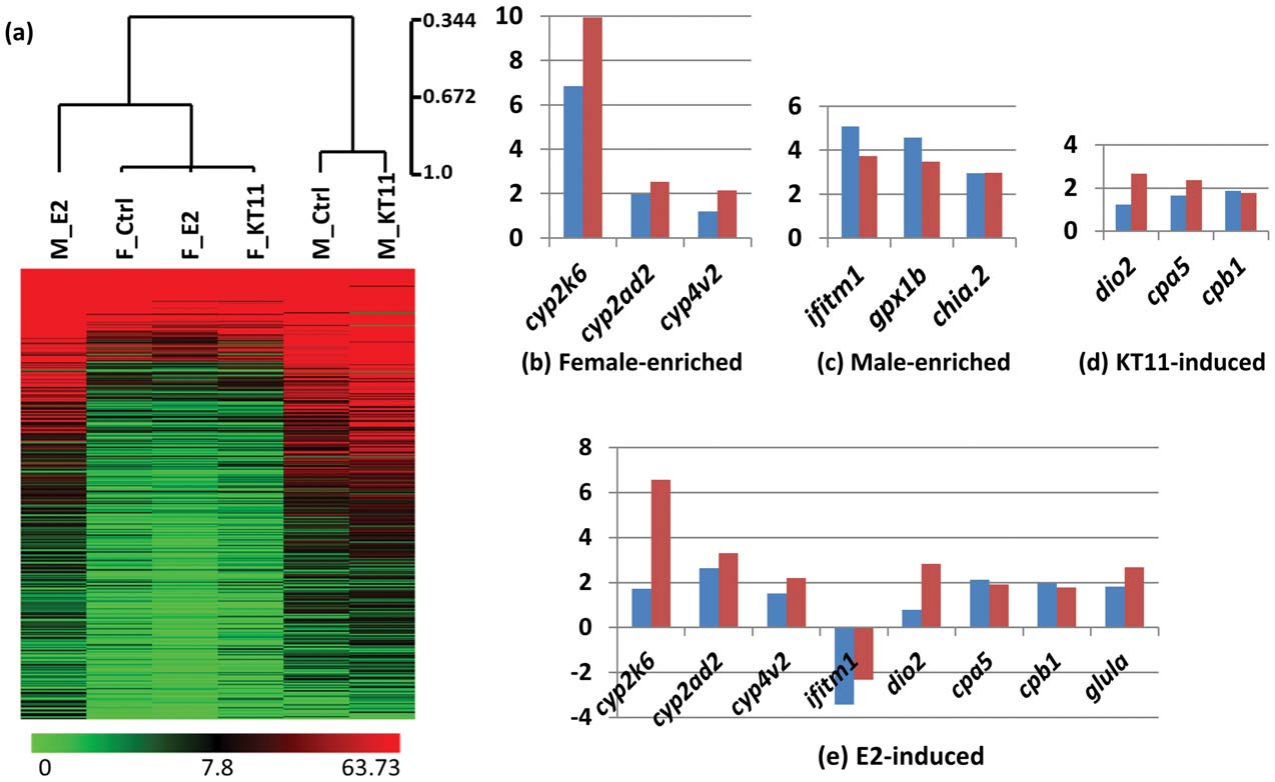
Hierarchical clustering of the six liver transcriptomes and real-time RT-PCR validation of RNA expression of selected genes. (a) Hierarchical clustering of the six liver transcriptomes from control males and females, E2-treated males and females, and KT11-treated males and females. The three female samples were closely clustered together, and E2-treated males was clustered with the female samples. Control males and KT11-treated males were clustered together and were distinct from the others. Scale bar represented the pearson correlation score. Heatmap was constructed with transcripts that showed significant differences in at least one comparison. (b–e) Real-time RT-PCR validation of transcripts enriched in the female liver (b), male liver (c), induced by KT11 (d) or E2 (e) in male livers. Fold changes (log2 base) measured by real-time RT-PCR (blue bars) are compared with those measured by RNA-SAGE sequencing (red bars).

Hierarchical clustering of the six samples showed that the three female samples were closely clustered together, indicating very minor transcriptome perturbations caused by E2 and KT11 treatments (**Figure 1a**). However, male liver treated with E2 was clustered with the female samples, suggesting that male liver after E2 treatment showed a higher similarity with the female liver. Control male liver and KT11-treated male liver were clustered together and were distinct from the others.

### Identification of Sex-biased Transcripts in the Zebrafish Liver

An MA plot was used to display differentially expressed genes between control male and female livers, where M-axis was defined as the logarithm-transformed fold change of expression levels for each gene and A-axis was defined as logarithm-transformed gene expression level for each gene. As illustrated in **Figure S1**, the log2-ratio-range of the differential gene expression levels between control female and male livers was decreased along M-axis when the gene expression levels were increased along A-axis, indicating that the fold change of gene expression levels should not be used as a sole significant indicator for identification of differentially expressed genes. This is because a small fold change of abundantly expressed genes can alter the transcriptome significantly while low abundant transcripts can only be considered significantly changed when having a higher fold change. Here we used edgeR to calculate p value based on both gene expression level and fold change and the cut-off was set at 0.05. Furthermore, we only included genes with expression level of at least 2 TPM in order to capture physiologically more relevant genes. Finally, we identified 186 female-biased transcripts and 121 male-biased transcripts (**Table S2**). Selected genes were analyzed by real-time RT-PCR from independent sets of fish samples and our data confirmed the accuracy of the RNA-SAGE sequencing (**Figure 1b and 1c**), as we previously reported in zebrafish using the same RNA-SAGE sequencing technology [**11**].

The female-biased gene list included vitellogenins and zona pellucida glycoproteins, many ribosomal proteins, and estrogen receptor 1 (*esr1*). It is noteworthy that a major difference between oviparous and viviparous females is the production of egg yolk proteins in the liver. In sexually mature females of oviparous animals, a large proportion of the liver energy is devoted to the synthesis of vitellogenins, which are specific glycolipo-phosphoproteins produced in the liver under estradiol stimulation, and transported to oocyte as maternal stored materials to support early development. In our transcriptome data, the 8 vitellogenin transcripts account for 78% of the transcripts in the female livers, while they make up only 0.3% of the male liver transcriptome. Zona pellucidas are membrane glycoproteins of the oocyte which are essential for oocyte fertilization [37]. Another prominent female biased gene, *nots* encodes an aspartic proteinase, which is synthesized in the liver and transported to the ovary [38], [39]. It has been reported as one of the most differentially up-regulated gene by E2 treatment [40] and is also up-regulated by polycyclic aromatic hydrocarbons and alkylphenols in female fish [41].

In contrast, the list of male-biased gene contains many liver-enriched transcripts, such as *fabp2*, apolipoprotein (*apoa4*), and complement factors (*f9b*, *f3b*). Several chitinase genes (*chia.2*, *chia.1*, and *chia.3*) also appeared in the list. The transcripts of these genes were either not identified in the female liver or at marginally detectable level. It is interesting to note that chitinase proteins have been previously identified in zebrafish ovarian follicle and testis, while the corresponding transcript counterparts have not been detected in fully-grown follicles or testis, suggesting that these proteins were synthesized in extraovarian and extratesticular tissues and transported to the gonads [39], [42]. Our transcriptome data provided evidence that chitinases were synthesized in the liver and the male fish might have higher synthetic activity of chitinases. The human homologs of chitinases are important in inflammatory processes and tissue remodeling during arthritis, asthma, and inflammatory bowel disease [43]. They may act similarly in the zebrafish reproductive organs in the anti-inflammatory defense.

### Transcriptomic Differences of the Female and Male Liver Transcriptome

To depict the functional constitutions of the sex-biased transcripts, the female- and male- biased transcripts were subjected to gene ontology analyses. As the female liver devotes a large portion of its energy to vitellogenesis, gene ontology analysis of the female-biased transcripts showed high enrichment of ribosomal proteins to support the active protein synthesis of vitellogenins and other maternal proteins to be deposited to the oocytes (**Table S3**). In contrast, the male-biased transcripts showed more diversity, including genes involved in carbohydrate, aminoglycan, polysaccharide and monosaccharide metabolism, chitin metabolism, blood coagulation pathway, protein transport and localization (**Table S3**).

**Figure 2:**
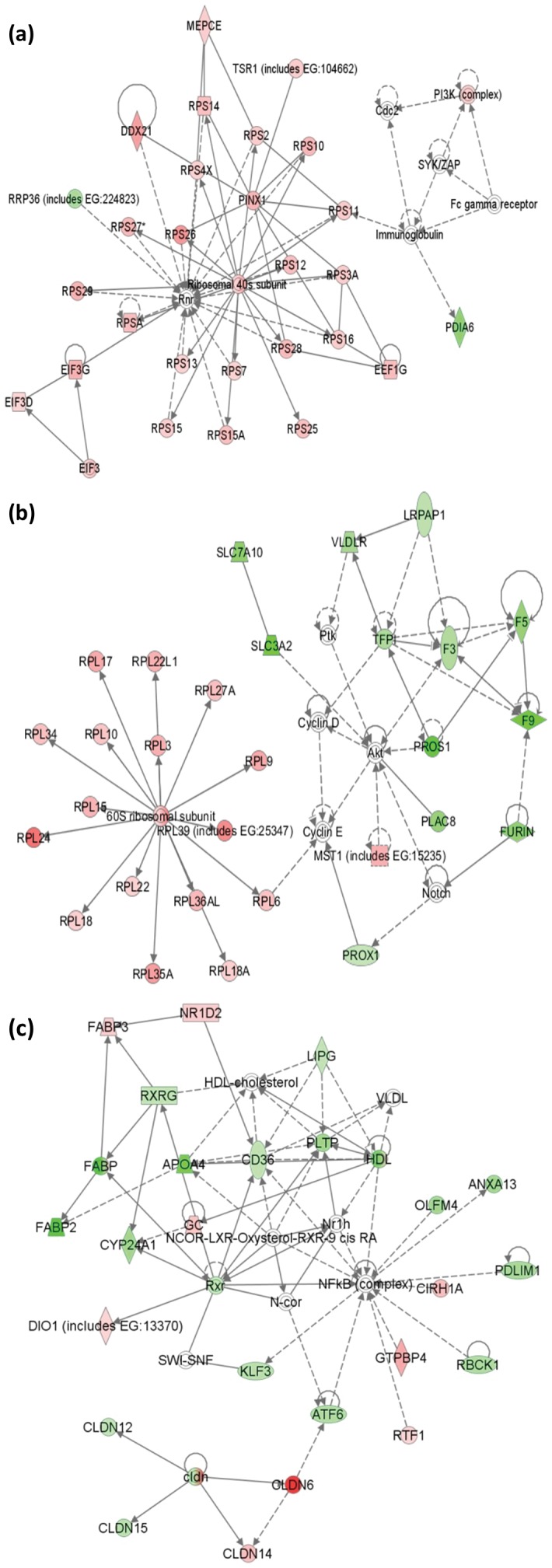
The top three networks as revealed by knowledge-based functional analyses of sex-biased genes. (a) The top network featured mainly by female-biased transcripts involving in gene expression, protein synthesis, RNA post-transcriptional modification (Score = 46). (b) The second network focuses on hematological system development and function, organismal functions, infectious disease (Score = 44). (c) The third network is associated with lipid metabolism, molecular transport, and small molecule biochemistry (Score = 39). Female-biased transcripts are indicated in red and male-biased transcripts in green.

To discover and visualize the biological connectivity of the sex-biased transcripts (p<0.1), the IPA network generating algorithm was used to maximize the interconnectedness of the transcripts based on all known connectivity in the database. The top two networks consisted mainly of only female- and male-biased transcripts and featured ribosome and coagulation. The top network is centered on the 40S ribosomal subunit with essentially exclusive female-biased transcripts (**Figure 2a**). The network with the second highest score featured the female-biased transcripts constituting the 40S ribosomal subunit and the male-biased transcripts involving in coagulation, fatty acid metabolism, and amino acid transport (**Figure 2b**). The third network was named as lipid metabolism, molecular transport, and small molecule biochemistry by IPA (**Figure 2c**). It is particularly interesting since it is consisted of transcripts from both sexes well interconnected, and it uncovered the central functions of several nuclear receptors, including NFkB, retinol x receptor (RXR) and liver x receptor (LXR). Particularly, RXR and LXR are critical regulators of the sex difference of liver transcriptome in terms of lipid and cholesterol metabolism [44], [45]. RXR participates in the regulation of cholesterol and fatty acid metabolism by interacting with other transcription regulators such as PPARG [46], which was also identified as activated in the male liver in the following transcription factor analysis. As indicated in the network in **Figure 2c**, female and male zebrafish liver has distinct expression pattern of claudin genes. The female liver is enriched with *cldn6* and *cldn14*, while the male liver is enriched with *cldn12* and *cldn15*. Claudins are critical components of tight junctions which regulate paracellular permeability and polarity. CLDN6 could be up-regulated by ERα activation [47], which is consistent with our observation that *cldn6* is highly female-biased.

### Sexual Dimorphism of Zebrafish Liver on Xenobiotic Metabolism and Anti-oxidation

It has been known that sexual dimorphism of the liver differentially affects the metabolism of drugs and toxins in both men and women [48]. CYPs are a large and diverse group of enzymes to catalyze the oxidation of organic substances and it has been found that the difference in CYP expression pattern is one of the major reasons resulting in differential drug metabolism between men and women [48], [49]. In the present study, we also identified several *cyp* transcripts biased either in the female or male zebrafish liver, including female-biased *cyp2k6*, *cyp2ad2*, *cyp4v2* and *cyp2aa4* and male-biased *cyp2aa2* and *cyp3c1l2*. Interestingly, all the four female-biased cyp genes were up-regulated in E2-treated male liver (**Table S4**), suggesting that these genes are E2-responsive. *Cyp2k6*, which is the most significant female-biased *cyp*, was demonstrated to catalyze the activation of the myco-toxin aflatoxin B1 (AFB1) to the carcinogenic exo-8,9-AFB1epoxide, and it is only expressed in the liver and ovary of the zebrafish [50]. It has been shown that the zebrafish is much more resistant to AFB1-induced carcinogenesis than the rainbow trout and the female fish is more susceptible to AFB1-induced DNA damage [51]. Our data probably provide a basis for this as male fish basically has no *cyp2k6* expression and they would be naturally resistant to AFB1, while female fish are more sensitive to AFB1 due to the higher level of *cyp2k6* expression.

Besides cyps, an antioxidant gene, *gpx1,* is expressed in male liver significantly higher than in female liver. Reactive oxygen species, such as superoxide and hydrogen peroxide, are generated in the mitochondrions and during enzymatic reactions. It can cause oxidative damage to DNA, proteins, and membrane lipids. Gpx1 is an intracellular antioxidant enzyme that enzymatically reduces hydrogen peroxide to water to limit its harmful effects. The higher level of *gpx1* in the male liver may be correlated with the higher mitochondrial activities and oxidoreduction. The gene encoding another enzyme which is known to reduce oxidative stress, *txnl4a*, was also enriched in the male liver.

### Differential Transcriptional Networks in the Female and Male Livers

**Figure 3:**
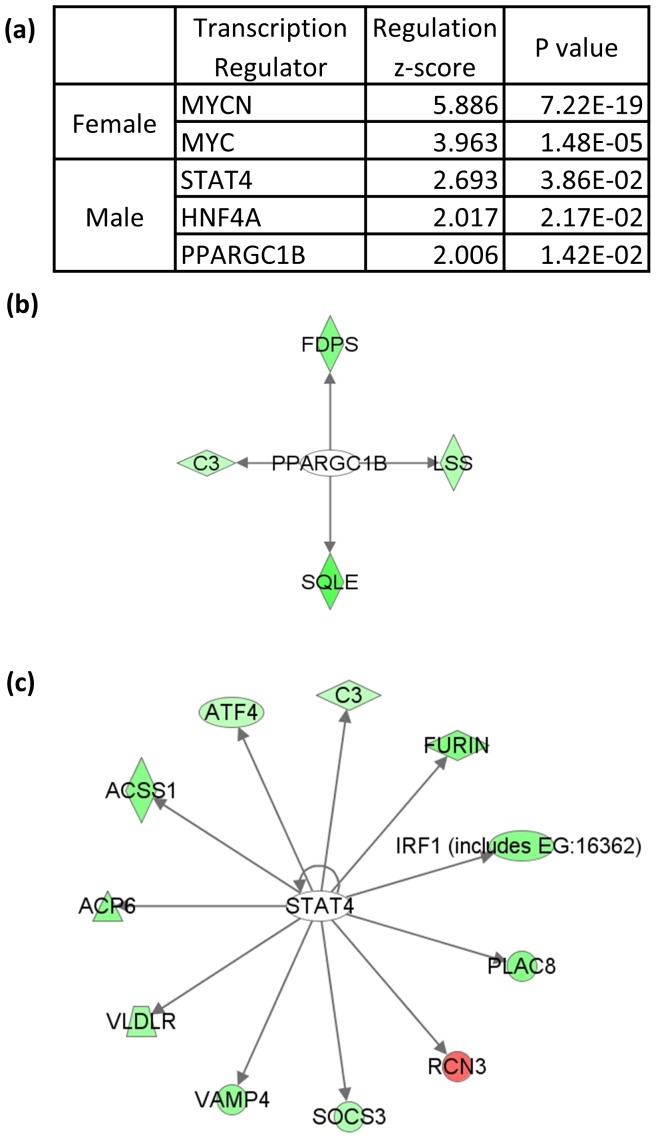
Significantly deregulated transcription factor networks. (a) List of sex-biased transcription factors in the zebrafish liver transcriptome. Regulation z-score indicates the degree of enrichment and p value indicates the level of significance. (b) Transcriptional targets network of *ppargc1b*. (c) Transcriptional targets network of *stat4*. Female- and male-biased genes are indicated in red and green, respectively. Non-colored genes are not in the sex-biased gene lists but are associated with the sex-biased genes and are introduced by the software to link up the network.

The intrinsic difference between female and male livers is also reflected by their different regulatory networks. Transcription factor (TF) analysis was conducted using female- and male-biased transcripts (p<0.1) to identify the transcription factors that may be responsible for the sex-biased gene expression profiles (**Figure 3a**). Different from previous methods which use predictive binding sites, IPA TF prediction is based on experimentally derived relationships from published literature, thus enabling to focus on important interactions with greater confidence in prediction. A transcription factor was predicted to be activated if z-score≥2 and inhibited if z-score≤-2, and the significance was defined by p value≤0.05. Our analysis indicated that Myc and Mycn are the only activated TFs in the female-biased transcripts. The majority of Myc-regulated female-biased transcripts are involved in ribosomal function [52]. Estrogen has been known to induce Myc expression by activating an upstream enhancer [53], [54], [55]. The activated TFs in male livers included Ppargc1b, Hnf4a and Stat4. Ppargc1b is involved in fat oxidation, non-oxidative glucose metabolism, and the regulation of energy expenditure [56], [57], [58] (**Figure 3b**). Hnf4a is best known as a master regulator of liver-specific gene expression, especially those involved in lipid transport and glucose metabolism [59], [60]. It has also been reported to contribute to the sex-specific expression of mouse liver *Cyp* genes by positively regulating male-specific genes [61]. The targets of Stat4 include Jak-Stat signaling transducer Irf1 and negative regulator Socs3 (**Figure 3c**). STATs (signal transducers and activators of transcription) are a family of TFs which could be activated by JAK (Janus kinase). The Jak-STAT signaling pathway has been implicated in a variety of cellular functions in the liver, such as liver metabolism and differentiation, antiviral defense in hepatitis virus infection, as well as liver injury and regeneration [62]. However, the function of Stat4 in liver is thus far poorly studied.

### Transcriptomic Changes Induced by Sex Hormone Treatment

The liver is highly sensitive to sex hormone disturbance. Previous studies have been conducted using either estrogen or androgen to examine the effect of sex hormone on liver transcriptome [40], [63], [64], [65], [66], [67], [68], [69], [70], this study is the first to directly compare the effect of estrogen and androgen in female and male. Genes whose expression levels have been significantly altered by E2 or KT11 treatment in the female or male zebrafish livers were identified using edgeR using cutoff at p value<0.05. The alterations of RNA expression of several genes were further confirmed by real-time PCR from independent RNA samples after the same sex hormone treatment (**Figure 1d and 1e**).

**Figure 4:**
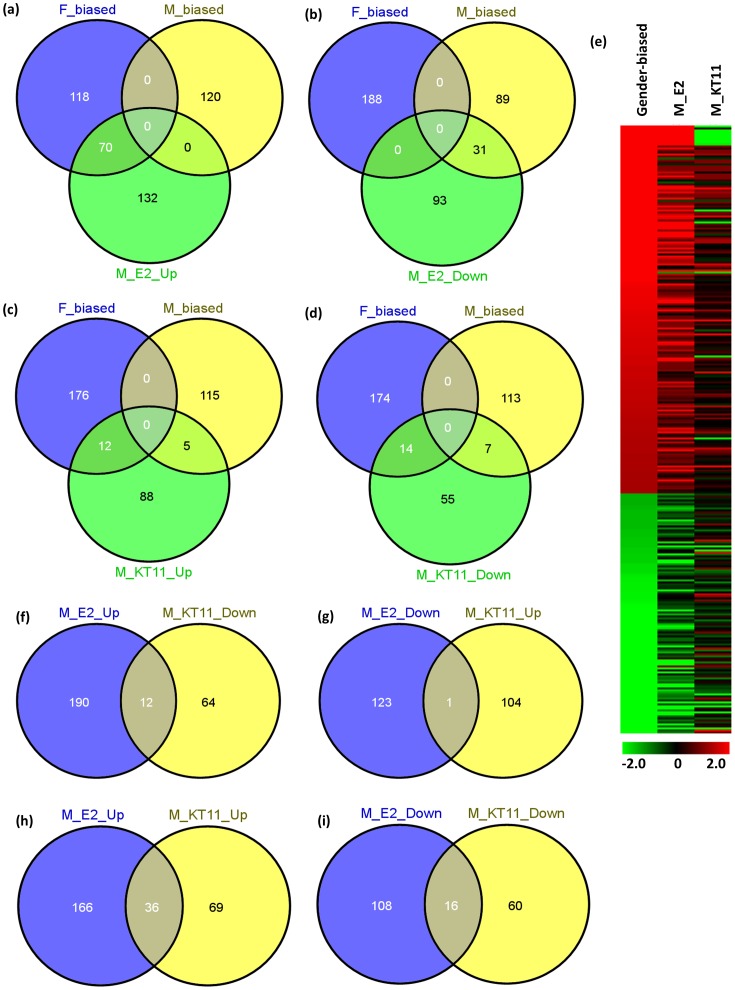
Intersections of differentially expressed genes in the male zebrafish livers after E2 or KT11 treatment with sex-biased genes. (a) Venn diagram of up-regulated genes in E2-treated males overlapped with sex-biased genes. (b) Venn diagram of down-regulated genes in E2-treated males overlapped with sex-biased genes. (c) Venn diagram of up-regulated genes in KT11-treated males overlapped with sex-biased genes. (d) Venn diagram of down-regulated genes in KT11-treated males overlapped with sex-biased genes . (e) Heat map of expression changes of sex-biased genes in E2- and KT11-treated male livers. For the column corresponding to the sex-biased transcripts, red represents female-biased genes and green represents male-biased genes. For the two columns corresponding to the E2- and KT11-treated male livers, red represents up-regulation and green represents down-regulation. The color intensity is calculated by logarithm-transformed (base 10) p-value. (f) Venn diagram of overlap of up-regulated genes in E2-treated male and down-regulated genes in KT11-treated males. (g) Venn diagram of overlap of down-regulated genes in E2-treated male and up-regulated genes in KT11-treated males. (h) Venn diagram of overlap of up-regulated genes in E2-treated male and up-regulated genes in KT11-treated males. (i) Venn diagram of overlap of down-regulated genes in E2-treated male and down-regulated genes in KT11-treated males.

Overall, the female zebrafish liver transcriptome did not show much change after E2 or KT11 treatment, as judged from a small number of genes significantly affected (**Table S4**). In E2-treated female livers, only five genes were up-regulated and 11 down-regulated. In KT11-treated female livers, 10 genes were up-regulated and 12 down-regulated. In contrast, both E2 and KT11 treatment induced dramatic transcriptome changes in male livers. In E2-treated male livers, 202 genes were up-regulated, 70 of which belonged to the female-biased transcripts and none of them was male-biased gene; 124 genes were down-regulated, 31 of which were male-biased transcripts and none of them was female-biased gene. Thus, 37.2% of female-biased transcripts and 25.8% of male-biased transcripts were responsive to E2 treatment of the male fish (**Figure 4a and 4b**), indicating that E2 treatment strongly feminized male liver. In the KT11-treated male liver, 105 transcripts were up-regulated and 76 transcripts were down-regulated. However, the overlapping of these differentially expressed genes with sex-biased genes was quite small (**Figure 4c and 4d**), suggesting that the KT11-induced liver transcriptomic changes are not sex-orientated. We also examined the change of all sex-biased transcripts in E2- and KT11-treated male livers (**Figure 4e**). The heat map showed that most of the female-biased transcripts were up-regulated (86.4%) and male-biased transcripts were down-regulated (58.3%) in the E2-treated male livers, while they did not show a very clear trend in the KT11-treated male livers. Furthermore, there were 12 genes up-regulated in E2-treated males but down-regulated in KT11-treated males (**Figure 4f**), including all the eight *vtg*s, *nots*, *gc*, *rpl39* and *rpl23a*. However, only one gene (*fabp6*) was down-regulated by E2 and up-regulated by 11KT (**Figure 4g**). Surprisingly, there were larger numbers of transcripts showing expression change at the same direction with either E2 or KT11 treatment; 36 genes were up-regulated by both E2 and KT11 (**Figure 4h**), and 16 genes were down-regulated by both E2 and KT11 (**Figure 4i**). These genes could be common targets of E2 and KT11 and several of these are involved in xenobiotic metabolism (*cpa1, cpa4, cpa5* and *cpb1*) and glutamate metabolism (*glub, sat2*).

E2 treatment up-regulated *vtg* expression in both female and male zebrafish livers. In the control female liver, the total number of *vtg* (*vtg1*–*7*) transcripts constitutes 78% of the transcriptome body. This high expression level was further up-regulated by E2 treatment to 85%. In the male liver, *vtg*s were only expressed at the level of 0.3 % of total transcriptome, while E2 treatment dramatically increased this by over 300 fold to 18.6%. Vitellogenin has been long recognized as a biomarker for environmental estrogenic compounds [71], [72]. However, *vtg* level in the female liver after KT11 treatment was not affected. Similar results have been reported by Hoffman *et. al.*, where female zebrafish treated with 17α-methyldihydrotestosterone (MDHT), a model androgen, showed no significant change in the level of *vtg* RNA [66]. In contrast to the female liver, the male liver is very sensitive to sex hormonal disturbance. Besides estrogenic compounds, the male liver could be feminized by various acute injuries [73], [74] and disruption of certain signaling pathways including Wnt signaling and the cytochrome P450 reductase system [75], [76]. Therefore, the male liver is predisposed to sexual dimorphic gene alterations in response to a wide variety of perturbations. It is also worthy to note that the current set of experiments employed fish directly from a local fish farm and these fish may had been exposed to a low level of estrogenic compounds from water and/or feed, as indicated by a relatively high level of *vtg* mRNAs in male control fish (**Table 1**). Nevertheless, we still observed a strong response of the male fish to E2 treatment in our current experiment.

**Table 1:** Top 50 expressed genes in the female and male livers.

	Female			Male		
	GI	Gene Symbol	TPM	GI	Gene Symbol	TPM
1	166795886	*vtg1*	475563.6	303304953	*fga*	117924.7
2	160420305	*vtg4*	188213.4	164698522	*il7r*	69007.6
3	68448529	*vtg5*	91869.6	227430294	*itln3*	26027.1
4	113678457	*vtg2*	24067.4	288856245	*LOC100003647*	25947.3
5	23308626	*fabp10a*	11708.9	292610076	*c3a*	22169.3
6	18859322	*rplp0*	8113.4	23308626	*fabp10a*	21888.8
7	62632716	*tfa*	7990.9	62632716	*tfa*	20925.9
8	303304953	*fga*	6546.7	292611118	*LOC100329302*	19307.8
9	41387125	*rps25*	4438.9	47085768	*fgg*	18839.7
10	164698522	*il7r*	4201.1	55925455	*gpx4a*	18297.0
11	47086524	*rps15a*	4142.4	166795886	*vtg1*	16449.5
12	41055645	*rps10*	3875.2	18859322	*rplp0*	12649.9
13	41055515	*rps27a*	3845.6	292626490	*LOC100334885*	11125.8
14	47271397	*rpl7*	3825.0	80751158	*zgc:123103*	10854.0
15	37700236	*tpt1*	3691.2	71834285	*apobl*	10589.9
16	47086132	*rps29*	3657.0	115529392	*zgc:152945*	9281.5
17	50540043	*rpl35a*	3525.7	189526818	*si:ch211-270n8.1*	8857.7
18	91176309	*rpl31*	3081.7	292621451	*a2m*	8826.5
19	51467910	*rpl6*	2970.3	18858246	*cfb*	8774.4
20	229335594	*rpl39*	2903.1	160420305	*vtg4*	8281.4
21	292610076	*c3a*	2869.9	47086792	*ahsg*	7600.9
22	41152198	*rps26l*	2712.4	221307487	*si:ch1073-126c3.2*	7303.7
23	80751158	*zgc:123103*	2704.1	41055515	*rps27a*	6890.0
24	47086792	*ahsg*	2623.0	41393104	*plg*	6781.0
25	226958508	*rps27.1*	2442.6	37700236	*tpt1*	6168.5
26	41053336	*rpl10*	2342.7	41387125	*rps25*	6006.3
27	48597013	*rpl3*	2314.2	47086524	*rps15a*	5513.9
28	41152463	*rps14*	1946.1	194578932	*apoa2*	5457.1
29	38564414	*mibp2*	1917.0	292622269	*apoc1l*	5214.0
30	55925455	*gpx4a*	1849.3	42734413	*igfbp1a*	4639.7
31	51010974	*rpl15*	1791.9	41055645	*rps10*	4622.5
32	189526818	*si:ch211-270n8.1*	1742.3	91176309	*rpl31*	4480.5
33	71834285	*apobl*	1724.2	31340752	*krt18*	4476.4
34	229335607	*rps28*	1669.9	300934727	*ambpl*	4421.7
35	41054304	*rpl5a*	1590.7	41152438	*rpl10a*	4332.7
36	50053845	*rps12*	1544.6	51467910	*rpl6*	4324.0
37	91176291	*rpl32*	1507.5	226958508	*rps27.1*	4133.9
38	160333704	*vtg2*	1476.9	47271397	*rpl7*	4085.6
39	292626490	*LOC100334885*	1457.1	121583580	*serpinf2b*	4047.3
40	292622269	*apoc1l*	1442.1	47086132	*rps29*	3994.1
41	61657912	*serp1*	1384.5	292622268	*LOC570585*	3894.7
42	288856245	*LOC100003647*	1378.8	38564414	*mibp2*	3870.6
43	61806481	*rps17*	1266.9	54261746	*ucp1*	3790.1
44	47085768	*fgg*	1255.5	41053336	*rpl10*	3596.7
45	292621451	*a2m*	1235.5	68448529	*vtg5*	3570.2
46	47086532	*ppia*	1126.1	162287364	*hpx*	3546.2
47	18858246	*cfb*	1103.2	50539723	*uox*	3452.2
48	194578932	*apoa2*	1062.8	40538763	*cp*	3441.9
49	50344933	*rpl11*	1020.0	113951770	*crp*	3213.3
50	41393104	*plg*	1013.9	33504508	*serpinc1*	3011.7

**Figure 5:**
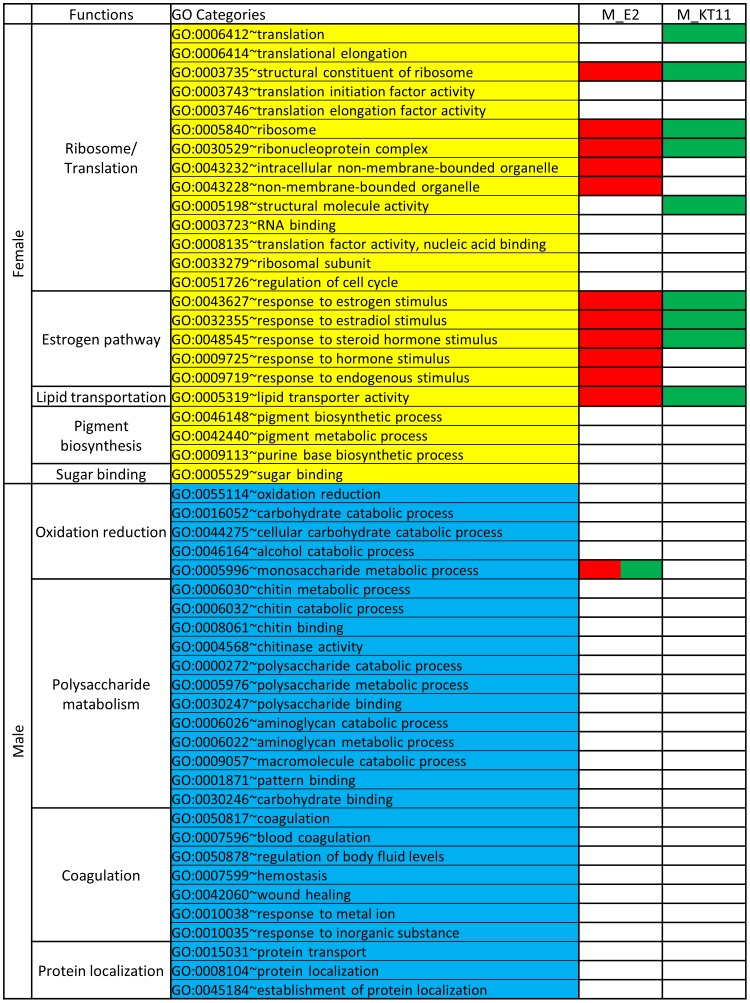
Changes of sex-biased GO categories in E2- and KT11-treated male zebrafish liver. Female-biased GO categories are in yellow shade and male-biased GO categories are in blue shade. GO categories under different classification (biological process, molecular function, and cellular component) are combined into functional categories to facilitate the interpretation. Red color represents up-regulation of the GO category and green color represents down-regulation of the GO category. It is clear that most of the female-biased GO categories were up-regulated in E2-treated males and down-regulated in KT11-treated males, while most of the male-biased GO categories were not affected.

As summarized in **Figure 5**, the female-biased genes are mainly involved in ribosome/translation, estrogen pathway, lipid transportation, pigment biosynthesis and sugar binding. In contrast, the male-biased genes are involved in Oxidation reduction, Polysaccharide metabolism, Coagulation and Protein localization. Gene ontology analysis of E2 up-regulated genes in male livers showed that the most abundant category was oxidation reduction, including several *cyps*, mitochondrial genes and various enzymes involved in carbohydrate, amino acid, and hydroxysteroid metabolisms (**Table S5**). In addition, many other prominent up-regulated categories were female-biased categories, such as response to estrogen stimulus, translation, and lipid transport (**Figure 5** **, Table S5**). Meanwhile, another small group of oxidoreduction-related transcripts was also down-regulated, including several *cyps*, heme xygenase (*hmox1*), eosinophil peroxidase (*epx*), and several enzymes involving in retinol and hydroxysteroid metabolisms. The bi-directional change of genes involved in oxidoreduction indicates that there were major shifts in the cellular metabolisms induced by E2 treatment.

Gene ontology enrichment analysis showed that up-regulated categories in KT11-treated male livers included proteolysis, immune response, lipid binding and catecholamine metabolic process (**Table S5**). Genes involved in immune response include major histocompatibility complex (MHC) genes and chemokines. In contrast, down-regulated categories were translation, lipid transport and response to estrogen stimulus, suggesting that KT11 further down-regulated female-biased categories (**Figure 5**). Interestingly, MHC genes showed both up- and down-regulation by KT11, indicating an alteration of antigen processing and presentation in the liver.

As indicated in our transcriptomic data, the effect of androgen treatment on the male livers was not sex-oriented. It is believed that liver masculinization is regulated predominantly by indirect androgen effects rather than by direct androgen receptor-ligand binding [77]. The sexual difference of liver transcriptome is controlled by the hypothalamic-pituitary-adrenal system. Catecholamines, especially epinephrine, play a crucial role in stress response and can modulate the basic function of immune cells in terms of proliferation, differentiation and production of cytokines. Catecholamines can influence the hepatic inflammatory response by altering hepatic blood flow through vasospasm and centrilobular hypoxia. Plasma catecholamine level is elevated in case of acute liver failure and acute and chronic hepatic inflammation [78]. Our result showed that administration of extra KT11 to the male fish up-regulated the catecholamine metabolism, which may provide a positive feedback cycle.

In summary, this study presented a comprehensive description of liver transcriptomes of the female and male zebrafish. We identified a list of sex-biased transcripts, which provides comprehensive information on sexual dimorphism of the zebrafish livers in term of functional categories, interactive networks and regulatory molecules. Our results from the sex hormone treatment experiments showed that the female zebrafish liver is relatively insensitive to sex hormone perturbation, while the male liver is highly responsive to both E2 and KT11 treatments. E2 feminizes the male liver by up-regulating many female biased genes that cause increase of vitellogenins synthesis and change of cellular metabolisms. In contrast, KT11 modulates the hypothalamic-pituitary-adrenal system, which imposes secondary effect on the liver.

## Supporting Information

Figure S1 0**MA plotting of the transcripts from control male and female samples.** M-axis is defined as the logarithm-transformed fold change of expression levels for each gene between control female and male liver while A-axis is defined as logarithm-transformed gene expression level for each gene. Red dots represented statistically significant differentially expressed genes between female and male liver (p-value<0.05).
(TIF)

Table S1 **Summary of RNA-SAGE sequencing results.**
(DOCX)

Table S2 **Lists of female- and male-biased genes in the zebrafish liver transcriptome.**
(DOCX)

Table S3 **Gene ontology enrichment analysis of female- and male-biased transcripts.**
(DOCX)

Table S4 **Lists of differently expressed transcripts in the female and male liver by E2 or KT11 treatment.**
(DOCX)

Table S5 **Gene ontology enrichment analysis of E2- and KT11-induced transcriptome changes in the male liver.**
(DOCX)
