# Supplementary figures and images for: Transcriptomic Analyses of Sexual Dimorphism of the Zebrafish Liver and the Effect of Sex Hormones
Source: PLoS One. 2013 Jan 17;8(1):e53562. doi: 10.1371/journal.pone.0053562 (PMC3547925; doi:10.1371/journal.pone.0053562)

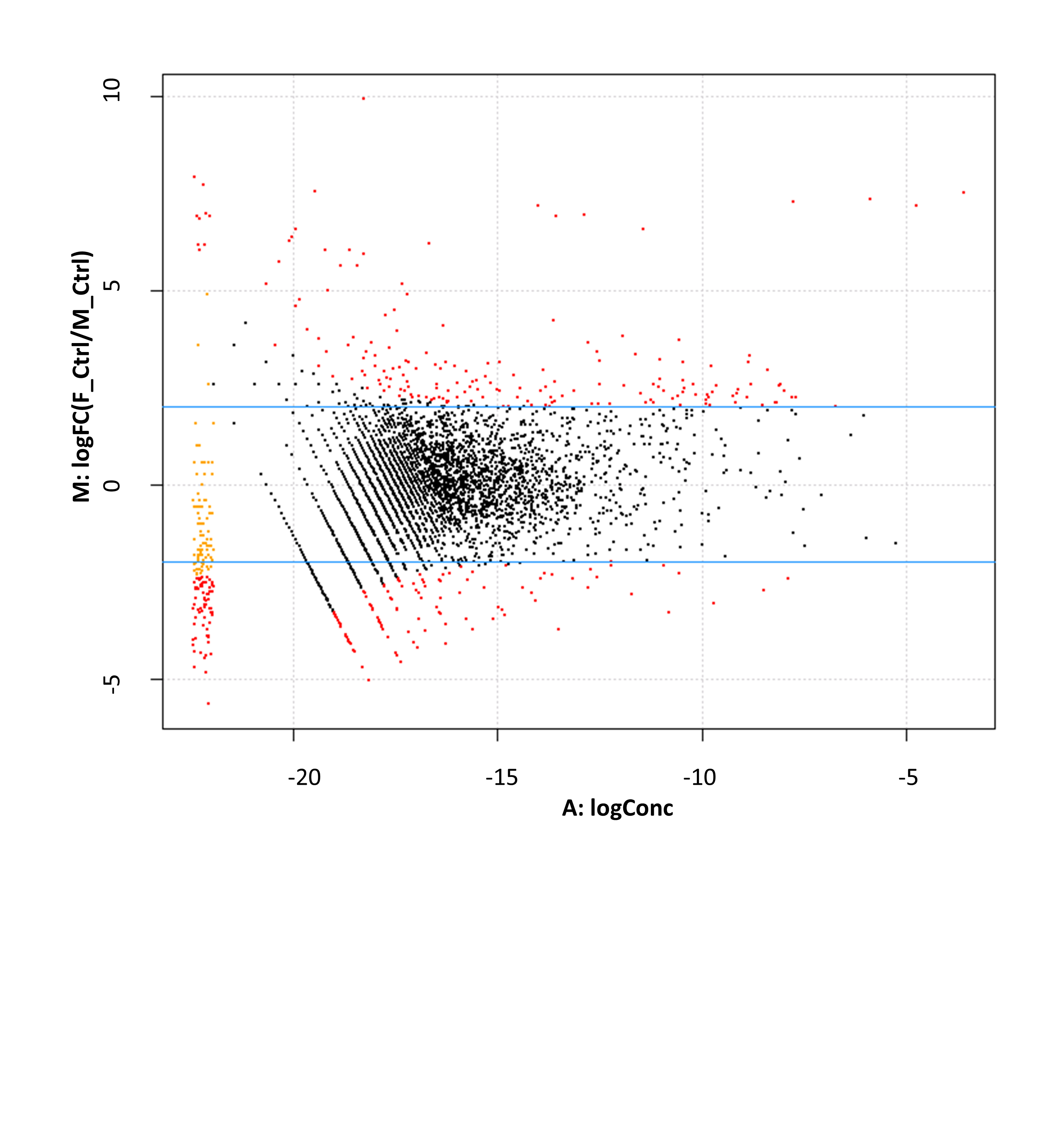

Supplement: Figure S1 — 0MA plotting of the transcripts from control male and female samples. M-axis is defined as the logarithm-transformed fold change of expression levels for each gene between control female and male liver while A-axis is defined as logarithm-transformed gene expression level for each gene. Red dots represented statistically significant differentially expressed genes between female and male liver (p-value<0.05). (TIF) [file pone.0053562.s001.tif]
