# Supplementary material for: Transcriptomic Analyses of Sexual Dimorphism of the Zebrafish Liver and the Effect of Sex Hormones
Source: PLoS One. 2013 Jan 17;8(1):e53562. doi: 10.1371/journal.pone.0053562 (PMC3547925; doi:10.1371/journal.pone.0053562)
Supplement: Table S1 — Summary of RNA-SAGE sequencing results. (DOCX) [file pone.0053562.s002.docx]

**Table S1. Summary of RNA-SAGE sequencing results**

| **Sample** | **Total reads** | **Total uniquely mapped reads** | **Mapping efficiency (%)** | **RefSeq entries** | **RefSeq entries above 30 tag counts** |
| --- | --- | --- | --- | --- | --- |
| F_Ctrl | 11,885,765 | 4,340,149 | 36.5 | 8,154 | 4,386 |
| F_E2 | 13,716,886 | 4,251,893 | 31.0 | 8,499 | 4,444 |
| F_11KT | 11,391,961 | 3,492,629 | 30.7 | 8,843 | 4,402 |
| M_Ctrl | 20,101,014 | 5,827,013 | 29.0 | 12,183 | 4,548 |
| M_E2 | 15,109,020 | 5,350,303 | 35.4 | 12,730 | 4,537 |
| M_11KT | 12,189,039 | 5,291,496 | 43.4 | 12,220 | 4,535 |
