# Supplementary material for: Transcriptomic Analyses of Sexual Dimorphism of the Zebrafish Liver and the Effect of Sex Hormones
Source: PLoS One. 2013 Jan 17;8(1):e53562. doi: 10.1371/journal.pone.0053562 (PMC3547925; doi:10.1371/journal.pone.0053562)
Supplement: Table S2 — Lists of female- and male-biased genes in the zebrafish liver transcriptome. (DOCX) [file pone.0053562.s003.docx]

**Table S2. Lists of female- and male-biased genes in the zebrafish liver transcriptome**

| **A. Female-biased genes** | | | | | | |
| --- | --- | --- | --- | --- | --- | --- |
| GI_ID | Gene | logConc | logFC | P.Value | F_Ctrl | M_Ctrl |
| 166795886 | *vtg1* | -5.96 | 2.78 | 4.82E-09 | 475563.6 | 16449.5 |
| 68448529 | *vtg5* | -5.88 | 7.34 | 8.24E-09 | 91869.6 | 3570.2 |
| 41055729 | *cyp2k6* | -18.26 | 9.93 | 8.36E-09 | 42.2 | 0.3 |
| 113678457 | *vtg2* | -7.78 | 7.28 | 1.01E-08 | 24067.4 | 976.9 |
| 160420305 | *vtg4* | -4.76 | 7.17 | 1.46E-08 | 188213.4 | 8281.4 |
| 156713466 | *vtg7* | -14.00 | 7.16 | 2.38E-08 | 310.2 | 13.7 |
| 303227888 | *vtg6* | -12.88 | 6.95 | 3.44E-08 | 625.5 | 32.0 |
| 292618717 | *vtg3* | -13.58 | 6.92 | 4.26E-08 | 381.2 | 19.9 |
| 160333704 | *vtg2* | -11.45 | 6.57 | 1.04E-07 | 1476.9 | 98.3 |
| 18859120 | *nots* | -16.67 | 6.19 | 1.93E-06 | 34.7 | 3.0 |
| 31341448 | *cldnd* | -32.69 | 34.65 | 5.84E-06 | 10.0 | 0.0 |
| 23308674 | *esr1* | -32.79 | 34.45 | 1.13E-05 | 8.8 | 0.0 |
| 292609855 | *si:ch73-90k17.1* | -19.46 | 7.53 | 1.68E-05 | 8.0 | 0.3 |
| 18859582 | *zp2* | -19.46 | 7.53 | 1.68E-05 | 8.0 | 0.3 |
| 292619383 | *LOC100331539* | -18.26 | 5.93 | 2.43E-05 | 10.6 | 1.1 |
| 41152033 | *pabpc4* | -17.33 | 5.16 | 3.45E-05 | 15.4 | 2.7 |
| 292627588 | *LOC795699* | -18.63 | 6.03 | 6.48E-05 | 8.5 | 0.8 |
| 154240693 | *si:ch211-250e5.16* | -18.42 | 5.62 | 6.48E-05 | 8.5 | 1.1 |
| 292609367 | *LOC100332229* | -17.20 | 4.90 | 7.08E-05 | 15.4 | 3.3 |
| 24158460 | *zp2.3* | -19.21 | 6.03 | 7.73E-05 | 5.7 | 0.5 |
| 157954493 | *zgc:174237* | -33.17 | 33.68 | 1.19E-04 | 5.1 | 0.0 |
| 156231015 | *retsatl* | -33.21 | 33.61 | 1.43E-04 | 4.9 | 0.0 |
| 118918375 | *si:ch211-14a17.7* | -33.21 | 33.61 | 1.43E-04 | 4.9 | 0.0 |
| 292609507 | *LOC557270* | -13.63 | 4.23 | 1.52E-04 | 145.2 | 48.9 |
| 156616359 | *zgc:174165* | -33.25 | 33.53 | 1.91E-04 | 4.6 | 0.0 |
| 50344971 | *rcn3* | -18.83 | 5.63 | 2.09E-04 | 6.4 | 0.8 |
| 292610747 | *buc* | -19.94 | 6.57 | 2.91E-04 | 4.1 | 0.3 |
| 118150575 | *npsn* | -17.53 | 4.47 | 3.57E-04 | 10.6 | 3.0 |
| 47086286 | *lgals3bpb* | -16.32 | 4.09 | 3.77E-04 | 21.4 | 7.9 |
| 71834591 | *reep2* | -17.74 | 4.34 | 3.92E-04 | 8.8 | 2.7 |
| 27545226 | *rpl24* | -11.95 | 3.82 | 4.52E-04 | 403.8 | 181.1 |
| 292616584 | *LOC793037* | -20.04 | 6.38 | 5.20E-04 | 3.6 | 0.3 |
| 50344933 | *rpl11* | -10.55 | 3.70 | 6.16E-04 | 1020.0 | 495.4 |
| 41055322 | *zgc:66313* | -12.77 | 3.65 | 7.25E-04 | 215.4 | 108.1 |
| 157841200 | *zgc:171776* | -20.09 | 6.27 | 7.67E-04 | 3.3 | 0.3 |
| 219277619 | *zgc:153499* | -20.09 | 6.27 | 7.67E-04 | 3.3 | 0.3 |
| 56693268 | *zgc:103559* | -17.45 | 3.94 | 1.08E-03 | 9.3 | 3.8 |
| 292618356 | [*im:7158796*](im:7158796) | -33.61 | 32.82 | 1.36E-03 | 2.8 | 0.0 |
| 292618151 | *LOC100332822* | -33.61 | 32.82 | 1.36E-03 | 2.8 | 0.0 |
| 292618149 | *LOC100332893* | -19.15 | 4.99 | 1.36E-03 | 4.1 | 0.8 |
| 295834927 | *LOC100333261* | -12.57 | 3.42 | 1.38E-03 | 229.6 | 135.2 |
| 292611593 | *LOC799637* | -11.61 | 3.36 | 1.61E-03 | 434.0 | 267.1 |
| 229335594 | *rpl39* | -8.85 | 3.31 | 1.80E-03 | 2903.1 | 1844.5 |
| 292625772 | *wu:fd14g04* | -33.67 | 32.68 | 2.21E-03 | 2.6 | 0.0 |
| 182509174 | *adh8a* | -11.02 | 3.20 | 2.46E-03 | 619.5 | 424.9 |
| 66773123 | *ela3l* | -12.51 | 3.18 | 2.65E-03 | 219.3 | 152.4 |
| 292628693 | *LOC100331622* | -18.08 | 3.64 | 2.87E-03 | 5.4 | 2.7 |
| 41152198 | *rps26l* | -8.86 | 3.14 | 2.89E-03 | 2712.4 | 1945.8 |
| 48597011 | *rpl23a* | -10.48 | 3.13 | 2.94E-03 | 882.1 | 635.0 |
| 45387524 | *lrrc17* | -16.73 | 3.38 | 3.10E-03 | 12.6 | 7.6 |
| 292614844 | *LOC100150687* | -20.36 | 5.74 | 3.14E-03 | 2.3 | 0.3 |
| 77993321 | *zgc:113028* | -19.86 | 4.74 | 3.14E-03 | 2.3 | 0.5 |
| 292624743 | *syne1a* | -17.66 | 3.52 | 3.42E-03 | 6.9 | 3.8 |
| 121583668 | *zgc:123178* | -14.96 | 3.14 | 3.51E-03 | 39.6 | 28.4 |
| 33504506 | *tgfbi* | -15.24 | 3.12 | 3.56E-03 | 32.4 | 23.5 |
| 61657912 | *zgc:92744* | -9.79 | 3.05 | 3.65E-03 | 1384.5 | 1054.7 |
| 50344951 | *rpp40l* | -18.54 | 3.77 | 3.78E-03 | 4.1 | 1.9 |
| 52218895 | *fkbp3* | -16.26 | 3.16 | 3.90E-03 | 16.2 | 11.5 |
| 47824884 | *mid1ip1* | -19.94 | 4.57 | 4.59E-03 | 2.1 | 0.5 |
| 50540043 | *rpl35a* | -8.38 | 2.93 | 4.96E-03 | 3525.7 | 2913.4 |
| 189526718 | *pprc1* | -18.20 | 3.42 | 5.11E-03 | 4.6 | 2.7 |
| 187960120 | *ddx21* | -13.88 | 2.95 | 5.13E-03 | 78.2 | 63.9 |
| 186910331 | *si:rp71-1g18.11* | -16.04 | 3.03 | 5.44E-03 | 18.0 | 13.9 |
| 57524558 | *zgc:101016* | -17.19 | 3.16 | 5.49E-03 | 8.5 | 6.0 |
| 50344911 | *fntb* | -16.52 | 3.08 | 5.63E-03 | 13.1 | 9.8 |
| 269914096 | *mastl* | -17.99 | 3.31 | 6.31E-03 | 5.1 | 3.3 |
| 160333616 | *prr16* | -17.25 | 3.18 | 6.33E-03 | 8.2 | 5.7 |
| 167621545 | *ddx43* | -18.63 | 3.57 | 6.45E-03 | 3.6 | 1.9 |
| 292621359 | *wu:fk33d07* | -18.63 | 3.57 | 6.45E-03 | 3.6 | 1.9 |
| 66472739 | *cpa1* | -15.77 | 2.91 | 6.59E-03 | 20.8 | 17.5 |
| 41053842 | *ccdc6b* | -16.39 | 2.97 | 6.99E-03 | 13.9 | 11.2 |
| 218931211 | *dtwd1* | -16.98 | 2.99 | 7.61E-03 | 9.3 | 7.4 |
| 56693266 | *sat2* | -14.60 | 2.80 | 8.00E-03 | 45.3 | 41.0 |
| 18859574 | *zp3b* | -18.28 | 3.25 | 8.09E-03 | 4.1 | 2.7 |
| 41053844 | *zgc:56041* | -17.40 | 3.01 | 8.22E-03 | 6.9 | 5.5 |
| 41054120 | *gtpbp4* | -11.04 | 2.71 | 8.84E-03 | 517.4 | 498.1 |
| 51467967 | *rpl9* | -15.29 | 2.77 | 9.33E-03 | 27.8 | 25.7 |
| 47086528 | *zgc:65996* | -10.46 | 2.67 | 9.81E-03 | 758.5 | 752.9 |
| 47087402 | *pla2g12b* | -13.61 | 2.65 | 1.10E-02 | 84.9 | 85.8 |
| 157743329 | *gamt* | -15.51 | 2.69 | 1.13E-02 | 23.2 | 22.7 |
| 50344867 | *rpl5b* | -11.18 | 2.59 | 1.21E-02 | 449.4 | 472.2 |
| 47087382 | *apex1* | -17.56 | 2.99 | 1.24E-02 | 6.2 | 4.9 |
| 41282216 | *eef1b2* | -10.22 | 2.57 | 1.24E-02 | 866.4 | 918.7 |
| 27545242 | *terfa* | -15.62 | 2.65 | 1.27E-02 | 21.1 | 21.3 |
| 47271397 | *rpl7* | -8.08 | 2.56 | 1.27E-02 | 3825.0 | 4085.6 |
| 48597013 | *rpl3* | -8.80 | 2.56 | 1.27E-02 | 2314.2 | 2473.2 |
| 41055905 | *eif3g* | -12.49 | 2.57 | 1.29E-02 | 179.4 | 191.2 |
| 157954495 | *zgc:173594* | -19.36 | 3.74 | 1.29E-02 | 2.3 | 1.1 |
| 118150455 | *zgc:153215* | -19.20 | 3.42 | 1.29E-02 | 2.3 | 1.4 |
| 61806481 | *zgc:114188* | -9.66 | 2.55 | 1.33E-02 | 1266.9 | 1368.8 |
| 47086132 | *rps29* | -8.13 | 2.53 | 1.38E-02 | 3657.0 | 3994.1 |
| 23308676 | *mst1* | -15.16 | 2.60 | 1.39E-02 | 28.6 | 29.8 |
| 23308680 | *cyp2ad2* | -11.91 | 2.53 | 1.41E-02 | 265.1 | 290.3 |
| 54400649 | *tpk1* | -11.05 | 2.50 | 1.50E-02 | 477.5 | 533.1 |
| 229324848 | *rpl22l1* | -16.00 | 2.60 | 1.51E-02 | 16.0 | 16.7 |
| 94536640 | *zgc:136871* | -18.01 | 3.04 | 1.57E-02 | 4.6 | 3.6 |
| 55742103 | *gyg1* | -13.83 | 2.50 | 1.60E-02 | 69.5 | 77.8 |
| 292612298 | *LOC100331950* | -17.30 | 2.80 | 1.61E-02 | 6.9 | 6.3 |
| 83699391 | *pinx1* | -17.78 | 2.90 | 1.65E-02 | 5.1 | 4.4 |
| 62751900 | *rpl12* | -10.43 | 2.46 | 1.66E-02 | 720.7 | 828.9 |
| 51010974 | *rpl15* | -9.11 | 2.45 | 1.69E-02 | 1791.9 | 2074.5 |
| 113679237 | *zgc:153662* | -15.68 | 2.52 | 1.74E-02 | 19.3 | 21.3 |
| 41053633 | *slc25a39* | -16.48 | 2.57 | 1.74E-02 | 11.3 | 12.0 |
| 41393174 | *eif4a1b* | -14.45 | 2.47 | 1.77E-02 | 44.8 | 51.1 |
| 45387526 | *smyd1a* | -19.65 | 3.99 | 1.84E-02 | 2.1 | 0.8 |
| 41055645 | *rps10* | -7.98 | 2.40 | 1.87E-02 | 3875.2 | 4622.5 |
| 148539931 | *si:dkey-51e6.1* | -13.95 | 2.41 | 1.92E-02 | 62.0 | 73.5 |
| 41152143 | *zgc:73262* | -10.93 | 2.40 | 1.92E-02 | 500.4 | 600.0 |
| 61806672 | *pop5* | -17.62 | 2.72 | 1.95E-02 | 5.4 | 5.2 |
| 41055903 | *tsc22d3* | -13.14 | 2.40 | 1.97E-02 | 107.8 | 128.9 |
| 57526754 | *rplp2* | -10.46 | 2.38 | 1.97E-02 | 688.8 | 832.9 |
| 41054514 | *pno1* | -15.02 | 2.43 | 1.99E-02 | 29.6 | 34.7 |
| 225007596 | *dkc1* | -15.47 | 2.44 | 1.99E-02 | 21.9 | 25.4 |
| 158534038 | *zgc:171429* | -17.21 | 2.63 | 2.04E-02 | 6.9 | 7.1 |
| 47086532 | *ppia* | -9.74 | 2.36 | 2.08E-02 | 1126.1 | 1384.3 |
| 50539811 | *ppp1r3ca* | -18.31 | 2.92 | 2.17E-02 | 3.6 | 3.0 |
| 41054745 | *gatm* | -18.25 | 2.80 | 2.17E-02 | 3.6 | 3.3 |
| 292610728 | *LOC100151188* | -11.52 | 2.34 | 2.19E-02 | 326.9 | 406.9 |
| 229335607 | *rps28* | -9.16 | 2.34 | 2.20E-02 | 1669.9 | 2087.8 |
| 40789251 | *rpl36a* | -14.94 | 2.39 | 2.20E-02 | 30.9 | 37.1 |
| 27545276 | *eef1g* | -9.88 | 2.30 | 2.38E-02 | 1000.7 | 1279.5 |
| 41056106 | *gmnn* | -17.70 | 2.73 | 2.40E-02 | 5.1 | 4.9 |
| 41054859 | *zgc:63631* | -16.22 | 2.41 | 2.41E-02 | 12.9 | 15.3 |
| 41393162 | *mmp13a* | -15.80 | 2.37 | 2.43E-02 | 17.0 | 20.8 |
| 94536911 | *zgc:136360* | -16.50 | 2.47 | 2.43E-02 | 10.8 | 12.3 |
| 41054971 | *rpsa* | -10.15 | 2.29 | 2.44E-02 | 830.3 | 1070.0 |
| 24119236 | *sec61al1* | -10.60 | 2.29 | 2.48E-02 | 604.6 | 782.7 |
| 50053845 | *rps12* | -9.24 | 2.28 | 2.51E-02 | 1544.6 | 2007.8 |
| 47086122 | *cirh1a* | -17.16 | 2.52 | 2.63E-02 | 6.9 | 7.6 |
| 292625234 | *LOC100334154* | -13.42 | 2.27 | 2.67E-02 | 84.9 | 111.2 |
| 168823557 | *zgc:171476* | -18.55 | 2.71 | 2.70E-02 | 2.8 | 2.7 |
| 18858740 | *gbp* | -18.55 | 2.71 | 2.70E-02 | 2.8 | 2.7 |
| 47086524 | *rps15a* | -7.80 | 2.25 | 2.72E-02 | 4142.4 | 5513.9 |
| 255069743 | *cldn2* | -17.66 | 2.50 | 2.77E-02 | 4.9 | 5.5 |
| 41152463 | *rps14* | -8.89 | 2.23 | 2.81E-02 | 1946.1 | 2617.1 |
| 62955122 | *zgc:110091* | -17.90 | 2.66 | 2.82E-02 | 4.4 | 4.4 |
| 50539709 | *trmt61a* | -17.85 | 2.57 | 2.82E-02 | 4.4 | 4.6 |
| 292619459 | *LOC563247* | -13.70 | 2.25 | 2.84E-02 | 69.5 | 92.3 |
| 47086130 | *rpl36* | -9.83 | 2.23 | 2.86E-02 | 1011.5 | 1366.3 |
| 41387125 | *rps25* | -7.69 | 2.22 | 2.87E-02 | 4438.9 | 6006.3 |
| 41053326 | *zgc:77235* | -10.71 | 2.20 | 3.04E-02 | 544.1 | 748.3 |
| 292625551 | *LOC795071* | -14.51 | 2.22 | 3.10E-02 | 39.4 | 53.3 |
| 55925471 | *eif4ebp3* | -17.27 | 2.40 | 3.15E-02 | 6.2 | 7.4 |
| 51010950 | *zgc:92868* | -11.26 | 2.18 | 3.16E-02 | 369.9 | 514.0 |
| 41055137 | *rpl34* | -9.91 | 2.18 | 3.20E-02 | 938.7 | 1312.0 |
| 292624112 | *si:ch73-15n24.1* | -15.35 | 2.24 | 3.27E-02 | 22.1 | 29.5 |
| 292625184 | *LOC100333127* | -15.97 | 2.25 | 3.45E-02 | 14.4 | 19.1 |
| 292622888 | [*im:6902407*](im:6902407) | -17.36 | 2.45 | 3.53E-02 | 5.9 | 6.8 |
| 223941794 | *si:ch211-217k17.7* | -13.59 | 2.14 | 3.59E-02 | 72.6 | 103.8 |
| 91176309 | *zgc:136896* | -8.17 | 2.12 | 3.63E-02 | 3081.7 | 4480.5 |
| 51467910 | *rpl6* | -8.22 | 2.12 | 3.65E-02 | 2970.3 | 4324.0 |
| 91176291 | *zgc:136952* | -9.19 | 2.11 | 3.72E-02 | 1507.5 | 2207.7 |
| 117606211 | *cyp4v2* | -14.25 | 2.14 | 3.74E-02 | 45.8 | 65.5 |
| 41393102 | *aldh9a1a* | -13.66 | 2.11 | 3.83E-02 | 68.5 | 100.0 |
| 149588618 | *ppat* | -17.11 | 2.32 | 3.87E-02 | 6.7 | 8.5 |
| 41054556 | *zgc:66382* | -11.20 | 2.09 | 3.89E-02 | 374.0 | 554.1 |
| 50540201 | *gc* | -11.39 | 2.09 | 3.94E-02 | 326.6 | 485.3 |
| 71892455 | *rrp12* | -16.34 | 2.22 | 3.96E-02 | 11.1 | 15.0 |
| 41055300 | *nat10* | -16.93 | 2.26 | 3.97E-02 | 7.5 | 9.8 |
| 121582335 | *si:rp71-15k1.1* | -12.27 | 2.08 | 4.00E-02 | 177.3 | 264.4 |
| 47085970 | *eif5a* | -9.89 | 2.07 | 4.03E-02 | 921.2 | 1382.4 |
| 113679532 | *si:ch211-117n7.7* | -17.77 | 2.41 | 4.04E-02 | 4.4 | 5.2 |
| 50344837 | *cyp2aa4* | -16.41 | 2.23 | 4.05E-02 | 10.6 | 14.2 |
| 47085790 | *caprin1b* | -12.53 | 2.08 | 4.08E-02 | 147.2 | 220.1 |
| 292620724 | *LOC559618* | -12.69 | 2.07 | 4.11E-02 | 131.8 | 198.0 |
| 130502098 | *zgc:162297* | -18.19 | 2.47 | 4.24E-02 | 3.3 | 3.8 |
| 154426307 | *rplp2l* | -9.81 | 2.05 | 4.29E-02 | 961.6 | 1470.9 |
| 224809426 | *dnttip2* | -16.54 | 2.20 | 4.29E-02 | 9.5 | 13.1 |
| 41053336 | *rpl10* | -8.52 | 2.04 | 4.33E-02 | 2342.7 | 3596.7 |
| 68448490 | *metap1* | -16.23 | 2.13 | 4.37E-02 | 11.6 | 16.7 |
| 113675127 | *si:ch211-199o1.5* | -15.65 | 2.13 | 4.40E-02 | 17.2 | 24.9 |
| 300795235 | *prkar1aa* | -19.04 | 2.77 | 4.40E-02 | 2.1 | 1.9 |
| 292627660 | *LOC100333875* | -16.61 | 2.18 | 4.43E-02 | 9.0 | 12.6 |
| 18859300 | *gnb2l1* | -10.52 | 2.03 | 4.44E-02 | 583.5 | 902.3 |
| 18859322 | *rplp0* | -6.72 | 2.02 | 4.54E-02 | 8113.4 | 12649.9 |
| 50345085 | *tfcp2l1* | -14.17 | 2.05 | 4.57E-02 | 46.8 | 71.6 |
| 292613814 | *LOC100148906* | -16.72 | 2.23 | 4.59E-02 | 8.5 | 11.5 |
| 56693256 | *zgc:101847* | -16.20 | 2.14 | 4.64E-02 | 11.8 | 16.9 |
| 47085914 | *zgc:64076* | -13.81 | 2.02 | 4.71E-02 | 59.5 | 92.6 |
| 18858728 | *gart* | -17.39 | 2.27 | 4.77E-02 | 5.4 | 7.1 |
| 54261764 | *psmg2* | -16.25 | 2.10 | 4.77E-02 | 11.3 | 16.7 |
| 47086176 | *zgc:85909* | -17.07 | 2.23 | 4.78E-02 | 6.7 | 9.0 |
| 72534834 | *pabpc1a* | -13.73 | 2.01 | 4.84E-02 | 62.8 | 98.6 |
| 38564422 | *wdr3* | -14.72 | 2.02 | 4.90E-02 | 31.7 | 49.4 |
| 65301455 | *nop10* | -14.84 | 2.02 | 4.91E-02 | 29.1 | 45.3 |

| **B. Male-biased genes** | | | | | | |
| --- | --- | --- | --- | --- | --- | --- |
| GI_ID | Gene | logConc | logFC | P.Value | F_Ctrl | M_Ctrl |
| 292609375 | *wu:fa56d06* | -32.51 | -35.01 | 8.72E-06 | 0.0 | 81.4 |
| 55742560 | *chia.2* | -32.92 | -34.20 | 9.09E-05 | 0.0 | 46.4 |
| 189521420 | *intl2* | -32.99 | -34.05 | 1.40E-04 | 0.0 | 42.1 |
| 110626158 | *fabp2* | -33.11 | -33.82 | 2.59E-04 | 0.0 | 35.8 |
| 292627692 | *LOC100331911* | -33.14 | -33.75 | 3.25E-04 | 0.0 | 34.1 |
| 47085760 | *chia.1* | -33.16 | -33.72 | 3.51E-04 | 0.0 | 33.3 |
| 116268038 | *zgc:154001* | -33.16 | -33.71 | 3.51E-04 | 0.0 | 33.0 |
| 115432033 | *zgc:153031* | -33.18 | -33.67 | 4.12E-04 | 0.0 | 32.2 |
| 57524618 | *chrne* | -33.26 | -33.50 | 6.30E-04 | 0.0 | 28.7 |
| 160333346 | *ifitm1* | -13.50 | -3.74 | 6.63E-04 | 10.0 | 844.7 |
| 189536130 | *si:ch211-66i15.5* | -16.28 | -4.11 | 6.71E-04 | 1.3 | 140.1 |
| 80751144 | *zgc:123218* | -18.14 | -5.03 | 7.39E-04 | 0.3 | 53.3 |
| 292613545 | *LOC100150202* | -33.30 | -33.43 | 7.55E-04 | 0.0 | 27.3 |
| 115529362 | *zgc:153027* | -17.37 | -4.56 | 8.12E-04 | 0.5 | 76.7 |
| 237820702 | *zgc:101744* | -16.97 | -4.20 | 9.23E-04 | 0.8 | 89.8 |
| 134133299 | *entpd3* | -33.34 | -33.34 | 1.01E-03 | 0.0 | 25.7 |
| 18858946 | *krt4* | -33.36 | -33.31 | 1.01E-03 | 0.0 | 25.1 |
| 292626310 | *LOC100149465* | -33.38 | -33.28 | 1.11E-03 | 0.0 | 24.6 |
| 292618008 | *LOC100334232* | -33.38 | -33.26 | 1.23E-03 | 0.0 | 24.3 |
| 18859380 | *slc3a2* | -33.39 | -33.25 | 1.23E-03 | 0.0 | 24.0 |
| 292618016 | *si:dkeyp-35f12.2* | -15.62 | -3.72 | 1.23E-03 | 2.3 | 193.4 |
| 83025077 | *zgc:123278* | -17.46 | -4.40 | 1.25E-03 | 0.5 | 68.5 |
| 94536957 | *f9b* | -17.03 | -4.07 | 1.33E-03 | 0.8 | 81.9 |
| 292611114 | *LOC566646* | -17.48 | -4.36 | 1.40E-03 | 0.5 | 66.6 |
| 123706193 | *slc39a3* | -18.30 | -4.70 | 1.75E-03 | 0.3 | 42.3 |
| 158517971 | *zgc:171801* | -33.48 | -33.07 | 1.89E-03 | 0.0 | 21.3 |
| 292622267 | *apoa4* | -15.12 | -3.45 | 1.97E-03 | 3.6 | 249.3 |
| 116267980 | *zgc:153628* | -16.76 | -3.78 | 2.36E-03 | 1.0 | 89.3 |
| 121582347 | *zgc:158432* | -33.53 | -32.98 | 2.38E-03 | 0.0 | 19.9 |
| 169636421 | *gpx1b* | -15.75 | -3.47 | 2.42E-03 | 2.3 | 162.2 |
| 168823461 | *zgc:171534* | -16.27 | -3.59 | 2.60E-03 | 1.5 | 117.4 |
| 41152043 | *pklr* | -17.16 | -3.81 | 2.63E-03 | 0.8 | 68.5 |
| 292622703 | *LOC100000736* | -33.54 | -32.96 | 2.68E-03 | 0.0 | 19.7 |
| 116875794 | *cyp2aa2* | -33.56 | -32.92 | 3.03E-03 | 0.0 | 19.1 |
| 292626424 | *LOC100003531* | -33.63 | -32.77 | 3.92E-03 | 0.0 | 17.2 |
| 55925318 | *cyp3c1l2* | -33.63 | -32.77 | 3.92E-03 | 0.0 | 17.2 |
| 292618210 | *grik1a* | -33.63 | -32.77 | 3.92E-03 | 0.0 | 17.2 |
| 292611524 | *LOC100332409* | -17.68 | -3.94 | 4.16E-03 | 0.5 | 50.0 |
| 148226073 | *zgc:163002* | -33.66 | -32.72 | 4.48E-03 | 0.0 | 16.7 |
| 52219039 | *dirc2* | -18.51 | -4.30 | 5.03E-03 | 0.3 | 32.0 |
| 27545262 | *atp6v0ca* | -33.69 | -32.65 | 5.14E-03 | 0.0 | 15.8 |
| 117606282 | *irg1l* | -33.69 | -32.65 | 5.14E-03 | 0.0 | 15.8 |
| 239582728 | *grn2* | -16.92 | -3.47 | 5.29E-03 | 1.0 | 72.1 |
| 157954501 | *zgc:171687* | -18.52 | -4.27 | 5.42E-03 | 0.3 | 31.4 |
| 45387834 | *ap1m2* | -33.71 | -32.62 | 5.92E-03 | 0.0 | 15.6 |
| 51011042 | *prr15la* | -33.72 | -32.60 | 5.92E-03 | 0.0 | 15.3 |
| 292618137 | *LOC795469* | -33.73 | -32.57 | 6.85E-03 | 0.0 | 15.0 |
| 55742576 | *zgc:65870* | -33.74 | -32.54 | 6.85E-03 | 0.0 | 14.7 |
| 45387592 | *npsnl* | -33.74 | -32.54 | 6.85E-03 | 0.0 | 14.7 |
| 76253692 | *zgc:113314* | -33.74 | -32.54 | 6.85E-03 | 0.0 | 14.7 |
| 68448476 | *isca1* | -33.74 | -32.54 | 6.85E-03 | 0.0 | 14.7 |
| 41056184 | *zgc:56585* | -17.79 | -3.72 | 7.36E-03 | 0.5 | 42.9 |
| 158517977 | *zgc:173927* | -33.76 | -32.52 | 7.96E-03 | 0.0 | 14.5 |
| 292615383 | *LOC100333117* | -18.60 | -4.11 | 8.01E-03 | 0.3 | 28.1 |
| 292616116 | *slc7a10* | -18.60 | -4.11 | 8.01E-03 | 0.3 | 28.1 |
| 41054124 | *zgc:63505* | -17.82 | -3.66 | 8.22E-03 | 0.5 | 41.2 |
| 70887562 | *fdps* | -17.83 | -3.65 | 8.71E-03 | 0.5 | 40.7 |
| 292622702 | *LOC100000596* | -33.80 | -32.43 | 9.31E-03 | 0.0 | 13.7 |
| 41054837 | *zdhhc16* | -33.80 | -32.43 | 9.31E-03 | 0.0 | 13.7 |
| 115529318 | *ugt5a2* | -33.80 | -32.43 | 9.31E-03 | 0.0 | 13.7 |
| 94536628 | *zgc:136439* | -33.80 | -32.43 | 9.31E-03 | 0.0 | 13.7 |
| 292611073 | *LOC100332407* | -18.63 | -4.06 | 9.46E-03 | 0.3 | 27.0 |
| 291049790 | *furinb* | -18.64 | -4.03 | 9.46E-03 | 0.3 | 26.5 |
| 71834671 | *miox* | -18.65 | -4.01 | 1.03E-02 | 0.3 | 26.2 |
| 164663831 | *si:dkey-98p3.7* | -17.87 | -3.57 | 1.04E-02 | 0.5 | 38.5 |
| 57525936 | *entpd1* | -33.84 | -32.34 | 1.09E-02 | 0.0 | 12.8 |
| 139947589 | *zgc:158845* | -17.89 | -3.52 | 1.17E-02 | 0.5 | 37.4 |
| 27545268 | *scarb2* | -18.69 | -3.93 | 1.23E-02 | 0.3 | 24.9 |
| 33636712 | *sult1st1* | -18.69 | -3.93 | 1.23E-02 | 0.3 | 24.9 |
| 50539903 | *zgc:92664* | -18.69 | -3.93 | 1.23E-02 | 0.3 | 24.9 |
| 47086016 | *chia.3* | -17.90 | -3.50 | 1.24E-02 | 0.5 | 36.9 |
| 18858408 | *cebpd* | -33.88 | -32.28 | 1.29E-02 | 0.0 | 12.3 |
| 189011587 | *zgc:174006* | -33.88 | -32.28 | 1.29E-02 | 0.0 | 12.3 |
| 45387902 | *irf11* | -33.88 | -32.28 | 1.29E-02 | 0.0 | 12.3 |
| 121583896 | *zgc:153955* | -33.89 | -32.25 | 1.29E-02 | 0.0 | 12.0 |
| 41055707 | *psmd3* | -33.89 | -32.25 | 1.29E-02 | 0.0 | 12.0 |
| 160333656 | *acp6* | -18.70 | -3.90 | 1.34E-02 | 0.3 | 24.3 |
| 47174757 | *ptgds* | -18.71 | -3.89 | 1.34E-02 | 0.3 | 24.0 |
| 41053793 | *zgc:77739* | -17.94 | -3.44 | 1.41E-02 | 0.5 | 35.2 |
| 41055226 | *chmp5* | -17.94 | -3.43 | 1.50E-02 | 0.5 | 35.0 |
| 40786526 | *erlec1* | -33.94 | -32.15 | 1.84E-02 | 0.0 | 11.2 |
| 41055248 | *zgc:55536* | -33.94 | -32.15 | 1.84E-02 | 0.0 | 11.2 |
| 41055455 | *zgc:63489* | -33.96 | -32.11 | 1.84E-02 | 0.0 | 10.9 |
| 148226167 | *si:dkey-12h9.7* | -33.98 | -32.07 | 1.84E-02 | 0.0 | 10.7 |
| 300934751 | *vamp4* | -33.98 | -32.07 | 1.84E-02 | 0.0 | 10.7 |
| 41055073 | *cbll1* | -34.00 | -32.04 | 2.21E-02 | 0.0 | 10.4 |
| 62955524 | *capslb* | -34.00 | -32.04 | 2.21E-02 | 0.0 | 10.4 |
| 189531238 | *LOC569381* | -34.02 | -32.00 | 2.21E-02 | 0.0 | 10.1 |
| 178057359 | *cmas* | -34.02 | -32.00 | 2.21E-02 | 0.0 | 10.1 |
| 57526688 | *pfkfb3* | -34.02 | -32.00 | 2.21E-02 | 0.0 | 10.1 |
| 292610233 | *LOC555374* | -18.83 | -3.66 | 2.40E-02 | 0.3 | 20.5 |
| 115497451 | *zgc:153138* | -18.83 | -3.66 | 2.40E-02 | 0.3 | 20.5 |
| 33504542 | *atp6v1ba* | -18.85 | -3.62 | 2.40E-02 | 0.3 | 19.9 |
| 169646704 | *vldlr* | -34.04 | -31.96 | 2.69E-02 | 0.0 | 9.8 |
| 292627475 | *si:dkey-197c15.6* | -34.04 | -31.96 | 2.69E-02 | 0.0 | 9.8 |
| 53749654 | *psmb9a* | -34.04 | -31.96 | 2.69E-02 | 0.0 | 9.8 |
| 292628115 | *LOC100332146* | -34.06 | -31.92 | 2.69E-02 | 0.0 | 9.6 |
| 41055007 | *pafah1b3* | -34.06 | -31.92 | 2.69E-02 | 0.0 | 9.6 |
| 292618979 | *LOC100003669* | -18.88 | -3.56 | 2.96E-02 | 0.3 | 19.1 |
| 148539920 | *cd164* | -18.90 | -3.51 | 2.96E-02 | 0.3 | 18.6 |
| 292621309 | *clca1* | -18.90 | -3.51 | 2.96E-02 | 0.3 | 18.6 |
| 62955712 | *pdlim1* | -18.90 | -3.51 | 2.96E-02 | 0.3 | 18.6 |
| 33504532 | *slc34a2b* | -34.08 | -31.88 | 3.29E-02 | 0.0 | 9.3 |
| 57525647 | *uevld* | -34.08 | -31.88 | 3.29E-02 | 0.0 | 9.3 |
| 292610016 | *LOC100331773* | -34.10 | -31.83 | 3.29E-02 | 0.0 | 9.0 |
| 115529338 | *znrf2* | -34.10 | -31.83 | 3.29E-02 | 0.0 | 9.0 |
| 48762666 | *col1a2* | -34.10 | -31.83 | 3.29E-02 | 0.0 | 9.0 |
| 121582337 | *si:dkey-236e20.6* | -34.10 | -31.83 | 3.29E-02 | 0.0 | 9.0 |
| 148277656 | *si:ch211-264f5.2* | -34.12 | -31.79 | 3.29E-02 | 0.0 | 8.7 |
| 292618977 | *eif4g3* | -34.12 | -31.79 | 3.29E-02 | 0.0 | 8.7 |
| 292625226 | *gtf3c5* | -18.91 | -3.49 | 3.30E-02 | 0.3 | 18.3 |
| 51591888 | *med23* | -18.91 | -3.49 | 3.30E-02 | 0.3 | 18.3 |
| 189530840 | *LOC794786* | -18.93 | -3.45 | 3.69E-02 | 0.3 | 17.8 |
| 54400403 | *txnl4a* | -18.94 | -3.43 | 3.69E-02 | 0.3 | 17.5 |
| 57524545 | *tnpo2* | -34.14 | -31.74 | 4.07E-02 | 0.0 | 8.5 |
| 33504530 | *tfpia* | -34.14 | -31.74 | 4.07E-02 | 0.0 | 8.5 |
| 262231808 | *ppp3ccb* | -34.14 | -31.74 | 4.07E-02 | 0.0 | 8.5 |
| 18858248 | *actc1b* | -34.17 | -31.69 | 4.07E-02 | 0.0 | 8.2 |
| 62955706 | *gnpda1* | -34.17 | -31.69 | 4.07E-02 | 0.0 | 8.2 |
| 62955432 | *f3b* | -18.95 | -3.40 | 4.14E-02 | 0.3 | 17.2 |
