# Supplementary material for: Transcriptomic Analyses of Sexual Dimorphism of the Zebrafish Liver and the Effect of Sex Hormones
Source: PLoS One. 2013 Jan 17;8(1):e53562. doi: 10.1371/journal.pone.0053562 (PMC3547925; doi:10.1371/journal.pone.0053562)
Supplement: Table S3 — Gene ontology enrichment analysis of female- and male-biased transcripts. (DOCX) [file pone.0053562.s004.docx]

**Table S3. Gene ontology enrichment analysis of female- and male-biased transcripts**

| **A. Enriched Gene Ontology terms in the female-biased transcripts** | | | | | |
| --- | --- | --- | --- | --- | --- |
|  | Term | Count | % | Fold Enrich | PValue |
| Biological Process | GO:0006412~translation | 38 | 24.84 | 12.32 | 1.27E-31 |
|  | GO:0006414~translational elongation | 5 | 3.27 | 24.8 | 3.74E-05 |
|  | GO:0051726~regulation of cell cycle | 6 | 3.92 | 10.54 | 2.28E-04 |
|  | GO:0043627~response to estrogen stimulus | 3 | 1.96 | 50.59 | 1.33E-03 |
|  | GO:0032355~response to estradiol stimulus | 3 | 1.96 | 50.59 | 1.33E-03 |
|  | GO:0048545~response to steroid hormone stimulus | 3 | 1.96 | 31.62 | 3.64E-03 |
|  | GO:0046148~pigment biosynthetic process | 3 | 1.96 | 21.08 | 8.31E-03 |
|  | GO:0009725~response to hormone stimulus | 3 | 1.96 | 15.81 | 1.47E-02 |
|  | GO:0042440~pigment metabolic process | 3 | 1.96 | 14.05 | 1.84E-02 |
|  | GO:0009719~response to endogenous stimulus | 3 | 1.96 | 13.31 | 2.04E-02 |
|  | GO:0009113~purine base biosynthetic process | 2 | 1.31 | 56.21 | 3.48E-02 |
| Molecular Function | GO:0003735~structural constituent of ribosome | 34 | 22.22 | 19.01 | 4.63E-34 |
|  | GO:0005198~structural molecule activity | 36 | 23.53 | 8.96 | 2.14E-24 |
|  | GO:0003723~RNA binding | 11 | 7.19 | 3.3 | 1.68E-03 |
|  | GO:0008135~translation factor activity, nucleic acid binding | 6 | 3.92 | 5.61 | 4.09E-03 |
|  | GO:0005529~sugar binding | 5 | 3.27 | 4.41 | 2.58E-02 |
|  | GO:0005319~lipid transporter activity | 3 | 1.96 | 11.34 | 2.77E-02 |
|  | GO:0003743~translation initiation factor activity | 4 | 2.61 | 5.77 | 3.10E-02 |
|  | GO:0003746~translation elongation factor activity | 3 | 1.96 | 8.82 | 4.42E-02 |
| Cellular Component | GO:0005840~ribosome | 36 | 23.53 | 15.54 | 6.89E-36 |
|  | GO:0030529~ribonucleoprotein complex | 38 | 24.84 | 10.48 | 1.32E-31 |
|  | GO:0043232~intracellular non-membrane-bounded organelle | 42 | 27.45 | 4.58 | 3.25E-21 |
|  | GO:0043228~non-membrane-bounded organelle | 42 | 27.45 | 4.58 | 3.25E-21 |
|  | GO:0019031~viral envelope | 2 | 1.31 | 48.93 | 3.97E-02 |
|  | GO:0005730~nucleolus | 4 | 2.61 | 5.06 | 4.19E-02 |
|  | GO:0033279~ribosomal subunit | 3 | 1.96 | 8.47 | 4.67E-02 |

| **B. Enriched Gene Ontology terms in the male-biased transcripts** | | | | | |
| --- | --- | --- | --- | --- | --- |
|  | Term | Count | % | Fold Enrich | PValue |
| Biological Process | GO:0016052~carbohydrate catabolic process | 7 | 7.53 | 15.97 | 3.70E-06 |
|  | GO:0006030~chitin metabolic process | 4 | 4.3 | 79.83 | 1.18E-05 |
|  | GO:0006032~chitin catabolic process | 4 | 4.3 | 79.83 | 1.18E-05 |
|  | GO:0000272~polysaccharide catabolic process | 4 | 4.3 | 49.13 | 5.91E-05 |
|  | GO:0006026~aminoglycan catabolic process | 4 | 4.3 | 49.13 | 5.91E-05 |
|  | GO:0005976~polysaccharide metabolic process | 5 | 5.38 | 19.96 | 9.61E-05 |
|  | GO:0006022~aminoglycan metabolic process | 4 | 4.3 | 24.56 | 5.08E-04 |
|  | GO:0009057~macromolecule catabolic process | 7 | 7.53 | 4.72 | 3.03E-03 |
|  | GO:0050817~coagulation | 3 | 3.23 | 20.83 | 8.57E-03 |
|  | GO:0007596~blood coagulation | 3 | 3.23 | 20.83 | 8.57E-03 |
|  | GO:0050878~regulation of body fluid levels | 3 | 3.23 | 20.83 | 8.57E-03 |
|  | GO:0007599~hemostasis | 3 | 3.23 | 20.83 | 8.57E-03 |
|  | GO:0005996~monosaccharide metabolic process | 4 | 4.3 | 7.51 | 1.50E-02 |
|  | GO:0010038~response to metal ion | 3 | 3.23 | 15.45 | 1.53E-02 |
|  | GO:0010035~response to inorganic substance | 3 | 3.23 | 11.68 | 2.59E-02 |
|  | GO:0055114~oxidation reduction | 8 | 8.6 | 2.53 | 3.23E-02 |
|  | GO:0015031~protein transport | 6 | 6.45 | 3.25 | 3.34E-02 |
|  | GO:0045184~establishment of protein localization | 6 | 6.45 | 3.25 | 3.34E-02 |
|  | GO:0042060~wound healing | 3 | 3.23 | 9.98 | 3.47E-02 |
|  | GO:0008104~protein localization | 6 | 6.45 | 3.05 | 4.19E-02 |
|  | GO:0044275~cellular carbohydrate catabolic process | 3 | 3.23 | 8.55 | 4.59E-02 |
|  | GO:0046164~alcohol catabolic process | 3 | 3.23 | 8.55 | 4.59E-02 |
| Molecular Function | GO:0008061~chitin binding | 4 | 4.3 | 110.03 | 3.90E-06 |
|  | GO:0004568~chitinase activity | 4 | 4.3 | 82.52 | 1.08E-05 |
|  | GO:0001871~pattern binding | 4 | 4.3 | 21.3 | 7.89E-04 |
|  | GO:0030247~polysaccharide binding | 4 | 4.3 | 21.3 | 7.89E-04 |
|  | GO:0030246~carbohydrate binding | 4 | 4.3 | 5.24 | 3.86E-02 |
| Cellular Component | GO:0005576~extracellular region | 9 | 9.68 | 2.88 | 7.75E-03 |
|  | GO:0005829~cytosol | 4 | 4.3 | 4.73 | 4.68E-02 |
