# Supplementary material for: Transcriptomic Analyses of Sexual Dimorphism of the Zebrafish Liver and the Effect of Sex Hormones
Source: PLoS One. 2013 Jan 17;8(1):e53562. doi: 10.1371/journal.pone.0053562 (PMC3547925; doi:10.1371/journal.pone.0053562)
Supplement: Table S4 — Lists of differently expressed transcripts in the female and male liver by E2 or KT11 treatment. (DOCX) [file pone.0053562.s005.docx]

**Table S4. Lists of differently expressed transcripts in the female and male liver by E2 or KT11 treatment**

| **A. E2 up-regulated genes in female livers** | | | | | | |
| --- | --- | --- | --- | --- | --- | --- |
| GI_ID | Gene | logConc | logFC | P.Value | DMSO_F | E2_F |
| 55742560 | *chia.2* | -32.45 | 35.14 | 1.23E-05 | 0.0 | 10.4 |
| 292609375 | *wu:fa56d06* | -32.97 | 34.08 | 3.94E-04 | 0.0 | 5.0 |
| 110626158 | *fabp2* | -32.97 | 34.08 | 3.94E-04 | 0.0 | 5.0 |
| 116875794 | *cyp2aa2* | -33.40 | 33.24 | 4.39E-03 | 0.0 | 2.8 |
| 18858946 | *krt4* | -33.62 | 32.79 | 1.32E-02 | 0.0 | 2.0 |

| **B. E2 down-regulated in female livers** | | | | | | |
| --- | --- | --- | --- | --- | --- | --- |
| GI_ID | Gene | logConc | logFC | P.Value | DMSO_F | E2_F |
| 118150575 | *npsn* | -32.66 | -34.70 | 5.70E-05 | 10.6 | 0.0 |
| 148277606 | *si:ch211-149p10.2* | -33.30 | -33.43 | 2.72E-03 | 4.4 | 0.0 |
| 41055579 | *sept7a* | -33.44 | -33.15 | 5.67E-03 | 3.6 | 0.0 |
| 47085910 | *lsm12* | -33.44 | -33.15 | 5.67E-03 | 3.6 | 0.0 |
| 292622073 | *atg12* | -33.49 | -33.05 | 7.41E-03 | 3.3 | 0.0 |
| 153791693 | *LOC554843* | -33.61 | -32.81 | 1.32E-02 | 2.8 | 0.0 |
| 130485902 | *zgc:162204* | -33.76 | -32.52 | 2.50E-02 | 2.3 | 0.0 |
| 47085926 | *arpc1b* | -33.84 | -32.35 | 3.55E-02 | 2.1 | 0.0 |
| 189521183 | *LOC100007087* | -33.84 | -32.35 | 3.55E-02 | 2.1 | 0.0 |
| 292611679 | *LOC100330261* | -33.84 | -32.35 | 3.55E-02 | 2.1 | 0.0 |
| 167555245 | *zgc:172352* | -33.84 | -32.35 | 3.55E-02 | 2.1 | 0.0 |

| **C. KT11 up-regulated genes in female livers** | | | | | | |
| --- | --- | --- | --- | --- | --- | --- |
| GI_ID | Gene | logConc | logFC | P.Value | F_Ctrl | F_KT11 |
| 292613907 | *si:dkey-46g23.2* | -32.75 | 34.52 | 8.20E-05 | 0.0 | 9.5 |
| 292626158 | *LOC100334908* | -32.92 | 34.19 | 2.46E-04 | 0.0 | 7.6 |
| 116875794 | *cyp2aa2* | -33.10 | 33.82 | 7.91E-04 | 0.0 | 5.9 |
| 48762666 | *col1a2* | -33.40 | 33.24 | 4.39E-03 | 0.0 | 3.9 |
| 47086700 | *cstf1* | -33.67 | 32.70 | 1.32E-02 | 0.0 | 2.7 |
| 292609375 | *wu:fa56d06* | -33.67 | 32.70 | 1.32E-02 | 0.0 | 2.7 |
| 41055007 | *pafah1b3* | -33.67 | 32.70 | 1.32E-02 | 0.0 | 2.7 |
| 45433523 | *klf3* | -33.67 | 32.70 | 1.32E-02 | 0.0 | 2.7 |
| 55742560 | *chia.2* | -33.74 | 32.56 | 1.80E-02 | 0.0 | 2.4 |
| 34098953 | *sst1.1* | -33.74 | 32.56 | 1.80E-02 | 0.0 | 2.4 |
| 57524545 | *tnpo2* | -33.90 | 32.24 | 3.55E-02 | 0.0 | 2.0 |

| **D. KT11 down-regulated genes in female livers** | | | | | | |
| --- | --- | --- | --- | --- | --- | --- |
| GI_ID | Gene | logConc | logFC | P.Value | DMSO_F | KT11_F |
| 56790259 | *ucp2* | -33.15 | -33.74 | 9.54E-04 | 5.4 | 0.0 |
| 189528136 | *LOC567149* | -33.26 | -33.52 | 1.75E-03 | 4.6 | 0.0 |
| 292621145 | *wu:fb61a09* | -33.26 | -33.52 | 1.75E-03 | 4.6 | 0.0 |
| 54400433 | *ap3s1* | -33.49 | -33.05 | 7.41E-03 | 3.3 | 0.0 |
| 292623166 | *LOC795096* | -33.49 | -33.05 | 7.41E-03 | 3.3 | 0.0 |
| 62955554 | *zgc:110346* | -33.61 | -32.81 | 1.32E-02 | 2.8 | 0.0 |
| 41152378 | *lgals9l1* | -33.61 | -32.81 | 1.32E-02 | 2.8 | 0.0 |
| 47086148 | *mrpl38* | -33.68 | -32.67 | 1.80E-02 | 2.6 | 0.0 |
| 41055541 | *gadd45aa* | -33.68 | -32.67 | 1.80E-02 | 2.6 | 0.0 |
| 190358635 | *si:ch211-71m22.1* | -33.76 | -32.52 | 2.50E-02 | 2.3 | 0.0 |
| 121582451 | *znf395a* | -33.76 | -32.52 | 2.50E-02 | 2.3 | 0.0 |
| 56693260 | *gcga* | -33.76 | -32.52 | 2.50E-02 | 2.3 | 0.0 |
| 292629235 | *LOC100334956* | -33.84 | -32.35 | 3.55E-02 | 2.1 | 0.0 |

| **E. E2 up-regulated genes in male livers** | | | | | | |
| --- | --- | --- | --- | --- | --- | --- |
| GI_ID | Gene | logConc | logFC | P.Value | M_DMSO | M_E2 |
| 292618717 | *vtg3* | -13.85 | 6.42 | 6.04E-09 | 19.9 | 1235.0 |
| 18859120 | *nots* | -16.46 | 6.66 | 9.20E-09 | 3.0 | 219.7 |
| 160333704 | *vtg2* | -11.87 | 5.76 | 6.19E-08 | 98.3 | 3853.6 |
| 156713466 | *vtg7* | -14.71 | 5.80 | 7.18E-08 | 13.7 | 548.0 |
| 303227888 | *vtg6* | -13.64 | 5.47 | 2.13E-07 | 32.0 | 1021.0 |
| 283436089 | *sc:d0261* | -19.78 | 6.93 | 1.57E-06 | 0.3 | 24.0 |
| 145587664 | *gbp4* | -13.52 | 4.84 | 2.24E-06 | 43.1 | 892.7 |
| 41055729 | *cyp2k6* | -19.96 | 6.57 | 5.79E-06 | 0.3 | 18.8 |
| 47086680 | *hmga2* | -18.65 | 5.19 | 1.32E-05 | 1.1 | 28.9 |
| 113678457 | *vtg2* | -9.39 | 4.11 | 3.00E-05 | 976.9 | 12148.5 |
| 113678962 | *zgc:153631* | -20.21 | 6.07 | 3.40E-05 | 0.3 | 13.3 |
| 47550784 | *pck1* | -15.34 | 3.96 | 6.73E-05 | 16.7 | 186.7 |
| 56693268 | *zgc:103559* | -17.38 | 4.12 | 7.10E-05 | 3.8 | 47.8 |
| 55925471 | *eif4ebp3* | -16.51 | 3.96 | 8.96E-05 | 7.4 | 82.8 |
| 23308674 | *esr1* | -34.00 | 32.03 | 9.30E-05 | 0.0 | 7.6 |
| 157743329 | *gamt* | -15.00 | 3.75 | 1.28E-04 | 22.7 | 220.2 |
| 292616584 | *LOC793037* | -20.42 | 5.65 | 1.35E-04 | 0.3 | 9.9 |
| 48762519 | *amy2a* | -17.32 | 3.86 | 1.63E-04 | 4.4 | 45.7 |
| 292619383 | *LOC100331539* | -19.04 | 4.42 | 1.97E-04 | 1.1 | 16.9 |
| 68448529 | *vtg5* | -7.79 | 3.56 | 2.05E-04 | 3570.2 | 30451.5 |
| 292623257 | *LOC556873* | -18.08 | 4.00 | 2.31E-04 | 2.5 | 28.4 |
| 47086296 | *hoga1* | -19.86 | 4.77 | 3.06E-04 | 0.5 | 10.7 |
| 45387754 | *zgc:77158* | -19.47 | 4.39 | 3.97E-04 | 0.8 | 12.4 |
| 292621440 | *LOC100329702* | -19.48 | 4.37 | 4.29E-04 | 0.8 | 12.2 |
| 167900489 | *gsdf* | -20.59 | 5.31 | 4.44E-04 | 0.3 | 7.8 |
| 186910331 | *si:rp71-1g18.11* | -15.87 | 3.40 | 4.68E-04 | 13.9 | 106.2 |
| 23308680 | *cyp2ad2* | -11.54 | 3.31 | 4.98E-04 | 290.3 | 2072.1 |
| 50344971 | *rcn3* | -19.50 | 4.32 | 5.03E-04 | 0.8 | 11.8 |
| 169403987 | *si:ch211-57h10.1* | -20.63 | 5.23 | 5.67E-04 | 0.3 | 7.4 |
| 292614844 | *LOC100150687* | -20.63 | 5.23 | 5.67E-04 | 0.3 | 7.4 |
| 295834927 | *LOC100333261* | -12.67 | 3.26 | 5.94E-04 | 135.2 | 935.2 |
| 218749804 | *acsf2* | -20.66 | 5.18 | 6.43E-04 | 0.3 | 7.2 |
| 292625198 | *LOC100334307* | -18.42 | 3.65 | 8.46E-04 | 2.2 | 19.8 |
| 292625589 | *cox8a* | -18.43 | 3.64 | 8.88E-04 | 2.2 | 19.6 |
| 160420305 | *vtg4* | -6.81 | 3.11 | 9.62E-04 | 8281.4 | 51441.6 |
| 292621034 | *c1qtnf2* | -20.04 | 4.42 | 9.92E-04 | 0.5 | 8.4 |
| 292626868 | *si:ch211-133j6.3* | -18.62 | 3.65 | 9.95E-04 | 1.9 | 17.3 |
| 47086598 | *hsd11b2* | -14.36 | 3.09 | 1.12E-03 | 44.2 | 272.0 |
| 292627402 | *LOC100330524* | -13.83 | 3.08 | 1.12E-03 | 64.2 | 390.6 |
| 41054919 | *pdia2* | -19.05 | 3.75 | 1.19E-03 | 1.4 | 13.3 |
| 70887704 | *slc5a6* | -18.83 | 3.66 | 1.22E-03 | 1.6 | 15.0 |
| 186910324 | *mych* | -17.19 | 3.19 | 1.30E-03 | 6.0 | 39.6 |
| 55925282 | *cyp2n13* | -15.21 | 3.02 | 1.49E-03 | 25.1 | 147.1 |
| 34098953 | *sst1.1* | -20.11 | 4.27 | 1.56E-03 | 0.5 | 7.6 |
| 238637258 | *igfbp6b* | -18.91 | 3.51 | 1.92E-03 | 1.6 | 13.5 |
| 292612298 | *LOC100331950* | -17.20 | 3.06 | 1.95E-03 | 6.3 | 37.7 |
| 166795886 | *vtg1* | -5.93 | 2.89 | 1.95E-03 | 16449.5 | 87856.3 |
| 77993321 | *zgc:113028* | -20.16 | 4.18 | 1.98E-03 | 0.5 | 7.2 |
| 139948744 | *ghrl* | -20.85 | 4.80 | 1.98E-03 | 0.3 | 5.5 |
| 47086594 | *mknk2a* | -16.08 | 2.94 | 2.10E-03 | 14.2 | 78.6 |
| 50540201 | *gc* | -11.03 | 2.84 | 2.26E-03 | 485.3 | 2513.5 |
| 169790921 | *dio2* | -12.20 | 2.84 | 2.31E-03 | 216.3 | 1117.0 |
| 54400459 | *fbln5* | -19.75 | 3.83 | 2.38E-03 | 0.8 | 8.4 |
| 41055785 | *mycb* | -14.90 | 2.86 | 2.41E-03 | 33.0 | 172.8 |
| 189517265 | *LOC555586* | -20.20 | 4.10 | 2.54E-03 | 0.5 | 6.7 |
| 157954495 | *zgc:173594* | -19.44 | 3.62 | 2.68E-03 | 1.1 | 9.7 |
| 189526718 | *pprc1* | -18.33 | 3.20 | 2.69E-03 | 2.7 | 18.1 |
| 41053633 | *slc25a39* | -16.34 | 2.89 | 2.73E-03 | 12.0 | 64.5 |
| 41054745 | *gatm* | -18.09 | 3.14 | 2.80E-03 | 3.3 | 20.9 |
| 164698524 | *ebi3* | -16.02 | 2.84 | 2.88E-03 | 15.3 | 78.8 |
| 160425212 | *si:dkey-239i20.2* | -17.16 | 2.90 | 3.11E-03 | 6.8 | 36.7 |
| 62955246 | *dctd* | -18.01 | 3.08 | 3.15E-03 | 3.6 | 21.7 |
| 68448470 | *cell* | -14.42 | 2.76 | 3.15E-03 | 47.8 | 233.4 |
| 50345093 | *zgc:77060* | -15.82 | 2.76 | 3.59E-03 | 18.0 | 88.3 |
| 189521687 | *LOC100151204* | -19.82 | 3.68 | 3.67E-03 | 0.8 | 7.6 |
| 31581592 | *glula* | -14.31 | 2.69 | 3.94E-03 | 52.7 | 245.2 |
| 45387524 | *lrrc17* | -17.05 | 2.78 | 4.29E-03 | 7.6 | 37.9 |
| 18858344 | *bcdo2l* | -18.85 | 3.17 | 4.32E-03 | 1.9 | 12.4 |
| 47824884 | *mid1ip1* | -20.30 | 3.90 | 4.34E-03 | 0.5 | 5.9 |
| 113679532 | *si:ch211-117n7.7* | -17.60 | 2.80 | 4.58E-03 | 5.2 | 26.1 |
| 50539811 | *ppp1r3ca* | -18.30 | 2.98 | 4.80E-03 | 3.0 | 17.1 |
| 61657912 | *zgc:92744* | -10.04 | 2.59 | 4.98E-03 | 1054.7 | 4573.1 |
| 27545226 | *rpl24* | -12.58 | 2.59 | 4.99E-03 | 181.1 | 787.5 |
| 41053767 | *slc25a32a* | -18.19 | 2.94 | 5.06E-03 | 3.3 | 18.1 |
| 189521220 | *cyp2r1* | -15.76 | 2.63 | 5.34E-03 | 19.7 | 87.9 |
| 157787160 | *zgc:174895* | -19.09 | 3.15 | 5.34E-03 | 1.6 | 10.5 |
| 66773123 | *ela3l* | -12.85 | 2.55 | 5.77E-03 | 152.4 | 642.2 |
| 131888315 | *ddb2* | -18.11 | 2.87 | 5.80E-03 | 3.6 | 18.8 |
| 41054500 | *zbtb16a* | -19.91 | 3.51 | 5.86E-03 | 0.8 | 6.7 |
| 160333616 | *prr16* | -17.51 | 2.68 | 6.34E-03 | 5.7 | 26.5 |
| 292627660 | *LOC100333875* | -16.44 | 2.56 | 6.71E-03 | 12.6 | 53.5 |
| 130507339 | *zgc:162239* | -18.77 | 2.95 | 6.99E-03 | 2.2 | 12.2 |
| 41054140 | *gnai2* | -19.34 | 3.17 | 7.02E-03 | 1.4 | 8.8 |
| 41054046 | *ela2l* | -12.04 | 2.47 | 7.20E-03 | 273.9 | 1092.6 |
| 55925461 | *dio1* | -15.69 | 2.50 | 7.42E-03 | 21.6 | 88.1 |
| 229335594 | *rpl39* | -9.30 | 2.45 | 7.51E-03 | 1844.5 | 7269.5 |
| 41055903 | *tsc22d3* | -13.14 | 2.45 | 7.71E-03 | 128.9 | 507.5 |
| 56693266 | *sat2* | -14.79 | 2.46 | 7.86E-03 | 41.0 | 162.9 |
| 47086638 | *degs1* | -17.49 | 2.59 | 8.10E-03 | 6.0 | 26.1 |
| 50878284 | *per1b* | -17.02 | 2.55 | 8.17E-03 | 8.5 | 35.8 |
| 48597011 | *rpl23a* | -10.87 | 2.39 | 8.92E-03 | 635.0 | 2404.3 |
| 33504506 | *tgfbi* | -15.61 | 2.42 | 9.20E-03 | 23.5 | 91.0 |
| 51556244 | *nr1d1* | -16.64 | 2.49 | 9.22E-03 | 11.2 | 45.5 |
| 40548301 | *cpa5* | -12.60 | 2.38 | 9.36E-03 | 191.4 | 719.1 |
| 118722329 | *prmt5* | -19.40 | 3.06 | 9.38E-03 | 1.4 | 8.2 |
| 118150455 | *zgc:153215* | -19.40 | 3.06 | 9.38E-03 | 1.4 | 8.2 |
| 41055081 | *rorab* | -18.06 | 2.56 | 9.90E-03 | 4.1 | 17.5 |
| 121583668 | *zgc:123178* | -15.35 | 2.40 | 9.99E-03 | 28.4 | 108.1 |
| 56693256 | *zgc:101847* | -16.08 | 2.42 | 1.01E-02 | 16.9 | 65.3 |
| 300795235 | *prkar1aa* | -19.02 | 2.84 | 1.06E-02 | 1.9 | 9.9 |
| 56790257 | *lpl* | -14.27 | 2.35 | 1.06E-02 | 60.9 | 224.2 |
| 41054556 | *zgc:66382* | -11.11 | 2.31 | 1.14E-02 | 554.1 | 1980.9 |
| 66472739 | *cpa1* | -16.06 | 2.37 | 1.15E-02 | 17.5 | 65.3 |
| 292625156 | *LOC566752* | -18.71 | 2.73 | 1.15E-02 | 2.5 | 11.8 |
| 41055322 | *zgc:66313* | -13.47 | 2.30 | 1.19E-02 | 108.1 | 384.9 |
| 47575846 | *mao* | -17.44 | 2.44 | 1.20E-02 | 6.6 | 25.7 |
| 70912383 | *tmem37* | -20.50 | 3.49 | 1.27E-02 | 0.5 | 4.4 |
| 189517091 | *sult3st1* | -19.72 | 3.05 | 1.28E-02 | 1.1 | 6.5 |
| 41054650 | *idh2* | -12.91 | 2.26 | 1.31E-02 | 161.4 | 559.4 |
| 292614157 | *LOC557176* | -14.85 | 2.28 | 1.32E-02 | 41.8 | 146.6 |
| 55925316 | *zgc:101797* | -19.05 | 2.78 | 1.36E-02 | 1.9 | 9.5 |
| 148230219 | *adrb2b* | -18.48 | 2.61 | 1.37E-02 | 3.0 | 13.3 |
| 41152198 | *rps26l* | -9.34 | 2.22 | 1.44E-02 | 1945.8 | 6560.7 |
| 269995932 | *urah* | -13.68 | 2.23 | 1.47E-02 | 96.1 | 324.5 |
| 51010980 | *cpa2* | -16.76 | 2.33 | 1.48E-02 | 10.9 | 39.6 |
| 50539947 | *decr1* | -16.06 | 2.28 | 1.48E-02 | 18.0 | 63.2 |
| 295834993 | *fetub* | -12.20 | 2.22 | 1.48E-02 | 268.7 | 901.1 |
| 57526754 | *rplp2* | -10.57 | 2.20 | 1.55E-02 | 832.9 | 2758.7 |
| 117606211 | *cyp4v2* | -14.24 | 2.20 | 1.59E-02 | 65.5 | 217.8 |
| 62955424 | *zgc:112160* | -16.20 | 2.24 | 1.65E-02 | 16.7 | 56.9 |
| 18858740 | *gbp* | -18.65 | 2.56 | 1.70E-02 | 2.7 | 11.6 |
| 18858372 | *btg2* | -16.03 | 2.21 | 1.78E-02 | 18.8 | 63.0 |
| 50540007 | *ldhbb* | -13.45 | 2.15 | 1.79E-02 | 115.5 | 371.2 |
| 292623930 | *wu:fd14a06* | -14.71 | 2.15 | 1.89E-02 | 48.3 | 154.4 |
| 45387526 | *smyd1a* | -20.15 | 3.03 | 1.97E-02 | 0.8 | 4.8 |
| 41053574 | *arl5c* | -15.33 | 2.14 | 2.01E-02 | 31.4 | 99.7 |
| 55925360 | *nosip* | -19.32 | 2.68 | 2.04E-02 | 1.6 | 7.6 |
| 47085692 | *pdcd4a* | -18.82 | 2.51 | 2.04E-02 | 2.5 | 10.1 |
| 182509174 | *adh8a* | -11.60 | 2.09 | 2.09E-02 | 424.9 | 1305.6 |
| 176866358 | *si:ch211-284o19.6* | -14.59 | 2.11 | 2.12E-02 | 53.0 | 164.8 |
| 150378484 | *LOC791723* | -18.57 | 2.44 | 2.13E-02 | 3.0 | 11.8 |
| 255683532 | *tdrd1* | -19.56 | 2.73 | 2.19E-02 | 1.4 | 6.5 |
| 41054478 | *zfp36l1b* | -14.44 | 2.08 | 2.23E-02 | 59.3 | 181.4 |
| 226823314 | *hsp90ab1* | -11.90 | 2.05 | 2.34E-02 | 350.1 | 1046.3 |
| 303227985 | *si:ch211-218c6.5* | -18.99 | 2.52 | 2.35E-02 | 2.2 | 9.1 |
| 48597013 | *rpl3* | -9.08 | 2.04 | 2.36E-02 | 2473.2 | 7361.1 |
| 55742600 | *zgc:92590* | -15.59 | 2.08 | 2.43E-02 | 26.8 | 81.5 |
| 269914096 | *mastl* | -18.48 | 2.37 | 2.44E-02 | 3.3 | 12.2 |
| 62955788 | *zgc:112992* | -19.59 | 2.68 | 2.46E-02 | 1.4 | 6.3 |
| 189521464 | *LOC407663* | -15.95 | 2.07 | 2.49E-02 | 21.0 | 63.6 |
| 116004500 | *nt5dc2* | -17.98 | 2.19 | 2.51E-02 | 4.9 | 16.2 |
| 292609507 | *LOC557270* | -14.74 | 2.04 | 2.51E-02 | 48.9 | 145.4 |
| 50345091 | *cpa4* | -19.17 | 2.54 | 2.53E-02 | 1.9 | 8.0 |
| 121583939 | *mettl7a* | -17.59 | 2.15 | 2.64E-02 | 6.6 | 21.1 |
| 225543277 | *zgc:92739* | -17.53 | 2.15 | 2.64E-02 | 6.8 | 21.9 |
| 41152200 | *skp1* | -16.05 | 2.06 | 2.69E-02 | 19.7 | 59.2 |
| 50540043 | *rpl35a* | -8.87 | 1.99 | 2.72E-02 | 2913.4 | 8359.5 |
| 292625551 | *LOC795071* | -14.63 | 2.01 | 2.75E-02 | 53.3 | 154.9 |
| 168823557 | *zgc:171476* | -18.75 | 2.36 | 2.78E-02 | 2.7 | 10.1 |
| 292610920 | *LOC100333432* | -17.15 | 2.11 | 2.79E-02 | 9.0 | 28.0 |
| 256355075 | *LOC568900* | -15.61 | 2.02 | 2.81E-02 | 27.0 | 79.2 |
| 41054424 | *eif4ebp3l* | -17.44 | 2.11 | 2.82E-02 | 7.4 | 23.0 |
| 41053844 | *zgc:56041* | -17.86 | 2.13 | 2.87E-02 | 5.5 | 17.3 |
| 148234349 | *isoc2* | -18.35 | 2.17 | 2.94E-02 | 3.8 | 12.4 |
| 94536961 | *bco2a* | -18.41 | 2.28 | 2.94E-02 | 3.6 | 12.4 |
| 41152446 | *hsdl2* | -14.19 | 1.97 | 3.00E-02 | 73.5 | 207.3 |
| 47086112 | *zgc:85829* | -14.99 | 1.98 | 3.03E-02 | 42.1 | 119.5 |
| 56693260 | *gcga* | -18.09 | 2.14 | 3.05E-02 | 4.6 | 14.7 |
| 167555177 | *zgc:172253* | -19.21 | 2.46 | 3.06E-02 | 1.9 | 7.6 |
| 121582335 | *si:rp71-15k1.1* | -12.35 | 1.95 | 3.07E-02 | 264.4 | 736.1 |
| 56090167 | *cel.1* | -12.22 | 1.94 | 3.10E-02 | 290.3 | 805.9 |
| 51556246 | *zgc:77395* | -16.75 | 2.01 | 3.13E-02 | 12.3 | 35.6 |
| 41054372 | *calrl2* | -15.88 | 1.98 | 3.16E-02 | 22.7 | 64.5 |
| 134133272 | *zgc:136564* | -17.62 | 2.08 | 3.17E-02 | 6.6 | 20.0 |
| 41282124 | *alas2* | -18.19 | 2.12 | 3.24E-02 | 4.4 | 13.7 |
| 50539781 | *hormad1* | -19.06 | 2.38 | 3.31E-02 | 2.2 | 8.2 |
| 41056122 | *pgam2* | -17.69 | 2.06 | 3.32E-02 | 6.3 | 19.0 |
| 47087432 | *eif4a2* | -16.95 | 2.02 | 3.35E-02 | 10.7 | 31.2 |
| 38198628 | *slc4a1a* | -18.54 | 2.24 | 3.36E-02 | 3.3 | 11.2 |
| 41055386 | *zgc:63663* | -19.43 | 2.47 | 3.43E-02 | 1.6 | 6.5 |
| 41393102 | *aldh9a1a* | -13.78 | 1.91 | 3.49E-02 | 100.0 | 270.5 |
| 94536678 | *ugt5b6* | -18.39 | 2.10 | 3.53E-02 | 3.8 | 11.8 |
| 225007596 | *dkc1* | -15.74 | 1.93 | 3.60E-02 | 25.4 | 69.7 |
| 41152005 | *pmp22a* | -19.25 | 2.38 | 3.71E-02 | 1.9 | 7.2 |
| 47087302 | *actr5* | -19.25 | 2.38 | 3.71E-02 | 1.9 | 7.2 |
| 50344837 | *cyp2aa4* | -16.58 | 1.93 | 3.77E-02 | 14.2 | 39.2 |
| 292624112 | *si:ch73-15n24.1* | -15.55 | 1.88 | 3.87E-02 | 29.5 | 78.6 |
| 50540263 | *sult1st6* | -16.48 | 1.92 | 3.87E-02 | 15.3 | 41.7 |
| 255069753 | *si:ch211-215l11.5* | -12.97 | 1.86 | 3.89E-02 | 178.3 | 467.1 |
| 50233770 | *nr1d2b* | -16.00 | 1.89 | 3.95E-02 | 21.6 | 57.7 |
| 37620195 | *rdh1* | -14.07 | 1.85 | 4.02E-02 | 83.0 | 216.4 |
| 41055901 | *pdk2* | -16.22 | 1.88 | 4.26E-02 | 18.6 | 49.3 |
| 66773119 | *zgc:112210* | -17.07 | 1.93 | 4.28E-02 | 10.1 | 27.8 |
| 35903022 | *mknk2b* | -18.08 | 1.99 | 4.29E-02 | 4.9 | 14.1 |
| 110626116 | *si:ch211-240l19.5* | -14.52 | 1.83 | 4.31E-02 | 61.4 | 158.0 |
| 192453575 | *klf9* | -16.25 | 1.87 | 4.35E-02 | 18.3 | 48.2 |
| 41056148 | *zgc:63850* | -17.20 | 1.92 | 4.46E-02 | 9.3 | 25.3 |
| 40363538 | *cyp4t8* | -16.13 | 1.86 | 4.48E-02 | 19.9 | 52.2 |
| 148540171 | *rspry1* | -19.30 | 2.29 | 4.54E-02 | 1.9 | 6.7 |
| 292621359 | *wu:fk33d07* | -19.30 | 2.29 | 4.54E-02 | 1.9 | 6.7 |
| 162138985 | *si:dkey-181m9.10* | -16.91 | 1.89 | 4.55E-02 | 11.5 | 30.8 |
| 41053874 | *cyp2k22* | -17.05 | 1.89 | 4.69E-02 | 10.4 | 27.8 |
| 50344933 | *rpl11* | -11.53 | 1.78 | 4.69E-02 | 495.4 | 1228.1 |
| 41152242 | *cdo1* | -17.95 | 1.94 | 4.72E-02 | 5.5 | 15.2 |
| 158517993 | *cpb1* | -11.83 | 1.78 | 4.75E-02 | 404.5 | 999.7 |
| 292620937 | *LOC100334215* | -19.13 | 2.23 | 4.76E-02 | 2.2 | 7.4 |
| 90017461 | *igf1* | -17.18 | 1.89 | 4.78E-02 | 9.6 | 25.5 |
| 41054354 | *ddx39aa* | -17.06 | 1.88 | 4.82E-02 | 10.4 | 27.6 |
| 61651721 | *mif4gdb* | -18.99 | 2.17 | 4.96E-02 | 2.5 | 8.0 |
| 47086842 | *brp44* | -13.75 | 1.77 | 4.96E-02 | 106.8 | 262.1 |
| 41054106 | *tdo2b* | -13.68 | 1.76 | 4.96E-02 | 112.2 | 275.2 |
| 94158181 | *zgc:136472* | -18.37 | 1.94 | 4.96E-02 | 4.1 | 11.4 |

| **F. E2 down-regulated genes in male livers** | | | | | | |
| --- | --- | --- | --- | --- | --- | --- |
| GI_ID | Gene | logConc | logFC | P.Value | M_DMSO | M_E2 |
| 160333606 | *zgc:172260* | -17.29 | -5.07 | 3.09E-06 | 98.3 | 2.1 |
| 125845146 | *nr0b2b* | -17.45 | -5.07 | 3.56E-06 | 88.2 | 1.9 |
| 292611114 | *LOC566646* | -17.83 | -5.03 | 6.08E-06 | 66.6 | 1.5 |
| 115529362 | *zgc:153027* | -17.47 | -4.72 | 1.14E-05 | 76.7 | 2.1 |
| 292630281 | *LOC100334439* | -19.35 | -5.60 | 1.26E-05 | 28.4 | 0.4 |
| 292613545 | *LOC100150202* | -19.38 | -5.55 | 1.55E-05 | 27.3 | 0.4 |
| 189536130 | *si:ch211-66i15.5* | -16.32 | -4.15 | 4.52E-05 | 140.1 | 5.7 |
| 57526004 | *pltp* | -18.82 | -4.67 | 5.89E-05 | 29.8 | 0.8 |
| 178057359 | *cmas* | -34.03 | -31.98 | 1.06E-04 | 10.1 | 0.0 |
| 292628115 | *LOC100332146* | -34.07 | -31.90 | 1.38E-04 | 9.6 | 0.0 |
| 47086492 | *mpx* | -14.43 | -3.66 | 1.67E-04 | 438.6 | 25.1 |
| 168823461 | *zgc:171534* | -16.35 | -3.70 | 2.03E-04 | 117.4 | 6.5 |
| 164698522 | *il7r* | -7.08 | -3.56 | 2.06E-04 | 69007.6 | 4216.4 |
| 41055179 | *hmgcs1* | -16.75 | -3.75 | 2.10E-04 | 90.1 | 4.8 |
| 292621145 | *wu:fb61a09* | -17.33 | -3.84 | 2.23E-04 | 62.5 | 3.2 |
| 192451476 | *samd7* | -19.80 | -4.71 | 2.80E-04 | 15.3 | 0.4 |
| 41055404 | *slc16a9a* | -15.16 | -3.49 | 3.28E-04 | 249.3 | 16.0 |
| 292610905 | *si:dkeyp-55c8.3* | -19.42 | -4.30 | 3.97E-04 | 17.2 | 0.6 |
| 134133299 | *entpd3* | -18.63 | -3.87 | 4.38E-04 | 25.7 | 1.3 |
| 123706193 | *slc39a3* | -17.71 | -3.48 | 6.45E-04 | 42.3 | 2.7 |
| 50539951 | *cldn15lb* | -16.48 | -3.25 | 8.93E-04 | 91.5 | 7.0 |
| 292627692 | *LOC100331911* | -17.99 | -3.41 | 9.41E-04 | 34.1 | 2.3 |
| 292623008 | *LOC561500* | -34.37 | -31.29 | 9.54E-04 | 6.3 | 0.0 |
| 62955622 | *zgc:110200* | -18.58 | -3.52 | 1.11E-03 | 23.5 | 1.5 |
| 157841229 | *cyp2aa7* | -17.16 | -3.21 | 1.36E-03 | 56.5 | 4.4 |
| 292622703 | *LOC100000736* | -18.82 | -3.49 | 1.47E-03 | 19.7 | 1.3 |
| 47087332 | *aqp3a* | -18.33 | -3.30 | 1.62E-03 | 25.9 | 1.9 |
| 189531238 | *LOC569381* | -20.09 | -4.11 | 1.75E-03 | 10.1 | 0.4 |
| 189530889 | *wu:fb66a12* | -17.02 | -3.09 | 1.75E-03 | 59.8 | 5.1 |
| 49227306 | *zgc:92326* | -16.92 | -2.96 | 2.51E-03 | 61.2 | 5.7 |
| 189521759 | *hp* | -11.79 | -2.79 | 2.65E-03 | 2016.3 | 209.8 |
| 292611073 | *LOC100332407* | -18.16 | -3.07 | 2.69E-03 | 27.0 | 2.3 |
| 62955554 | *zgc:110346* | -17.95 | -3.01 | 2.90E-03 | 30.6 | 2.7 |
| 54400589 | *cldn5b* | -19.17 | -3.31 | 3.45E-03 | 14.5 | 1.1 |
| 157311774 | *si:ch211-137i24.10* | -16.03 | -2.79 | 3.46E-03 | 106.8 | 11.2 |
| 292619623 | *wu:fd60h05* | -18.45 | -3.06 | 3.55E-03 | 21.8 | 1.9 |
| 189011587 | *zgc:174006* | -19.45 | -3.40 | 3.55E-03 | 12.3 | 0.8 |
| 41055073 | *cbll1* | -19.78 | -3.57 | 3.67E-03 | 10.4 | 0.6 |
| 292614630 | *si:dkey-52h23.1* | -19.80 | -3.53 | 4.11E-03 | 10.1 | 0.6 |
| 189521420 | *intl2* | -17.41 | -2.85 | 4.24E-03 | 42.1 | 4.2 |
| 41054398 | *magt1* | -17.48 | -2.86 | 4.34E-03 | 40.1 | 4.0 |
| 113673423 | *zgc:153650* | -19.50 | -3.30 | 4.78E-03 | 11.5 | 0.8 |
| 292617855 | *LOC794053* | -20.27 | -3.76 | 5.00E-03 | 7.9 | 0.4 |
| 47085886 | *marcksl1* | -17.90 | -2.89 | 5.07E-03 | 30.3 | 2.9 |
| 76253862 | *adrm1b* | -17.00 | -2.69 | 5.54E-03 | 52.7 | 5.9 |
| 221307487 | *si:ch1073-126c3.2* | -9.80 | -2.52 | 6.02E-03 | 7303.7 | 916.7 |
| 292618210 | *grik1a* | -18.71 | -2.88 | 6.53E-03 | 17.2 | 1.7 |
| 292626424 | *LOC100003531* | -18.71 | -2.88 | 6.53E-03 | 17.2 | 1.7 |
| 83025065 | *zgc:123282* | -18.19 | -2.75 | 6.78E-03 | 23.5 | 2.5 |
| 292614171 | *im:7138837* | -14.69 | -2.47 | 7.55E-03 | 243.1 | 31.6 |
| 90093315 | *slc43a3b* | -14.38 | -2.45 | 7.83E-03 | 299.0 | 39.4 |
| 157954501 | *zgc:171687* | -17.73 | -2.66 | 8.36E-03 | 31.4 | 3.6 |
| 292625448 | *LOC564720* | -19.11 | -2.90 | 8.81E-03 | 13.1 | 1.3 |
| 66472535 | *casp7* | -19.36 | -2.94 | 9.38E-03 | 11.2 | 1.1 |
| 55925318 | *cyp3c1l2* | -18.63 | -2.71 | 9.41E-03 | 17.2 | 1.9 |
| 139947599 | *zgc:162509* | -21.08 | -4.15 | 9.79E-03 | 5.2 | 0.2 |
| 42734413 | *igfbp1a* | -10.37 | -2.35 | 9.97E-03 | 4639.7 | 655.7 |
| 189514628 | *adamtsl7* | -18.51 | -2.65 | 1.01E-02 | 18.3 | 2.1 |
| 292615383 | *LOC100333117* | -17.86 | -2.59 | 1.05E-02 | 28.1 | 3.4 |
| 160333346 | *ifitm1* | -12.81 | -2.31 | 1.16E-02 | 844.7 | 123.3 |
| 41054706 | *cd63* | -13.13 | -2.30 | 1.19E-02 | 675.1 | 99.0 |
| 292626158 | *LOC100334908* | -21.11 | -4.07 | 1.21E-02 | 4.9 | 0.2 |
| 121582337 | *si:dkey-236e20.6* | -19.68 | -2.95 | 1.28E-02 | 9.0 | 0.8 |
| 42476242 | *odc1* | -19.02 | -2.65 | 1.36E-02 | 12.8 | 1.5 |
| 148233681 | *zgc:162351* | -19.43 | -2.79 | 1.41E-02 | 10.1 | 1.1 |
| 292615976 | *LOC560548* | -14.19 | -2.25 | 1.42E-02 | 317.3 | 48.2 |
| 50539975 | *dgat1b* | -16.94 | -2.33 | 1.47E-02 | 48.3 | 7.0 |
| 41054006 | *yipf3* | -16.58 | -2.30 | 1.56E-02 | 61.7 | 9.1 |
| 169646704 | *vldlr* | -19.45 | -2.75 | 1.57E-02 | 9.8 | 1.1 |
| 139948262 | *si:dkey-102c8.6* | -16.84 | -2.27 | 1.64E-02 | 50.8 | 7.6 |
| 167621450 | *rpa1* | -18.88 | -2.55 | 1.69E-02 | 13.7 | 1.7 |
| 116004520 | *f13a1* | -18.88 | -2.55 | 1.69E-02 | 13.7 | 1.7 |
| 148231475 | *si:ch211-154o6.6* | -12.51 | -2.17 | 1.69E-02 | 988.3 | 158.4 |
| 50539717 | *rdh12* | -17.80 | -2.36 | 1.81E-02 | 27.0 | 3.8 |
| 292616116 | *slc7a10* | -17.73 | -2.34 | 1.84E-02 | 28.1 | 4.0 |
| 57768902 | *zgc:101000* | -17.42 | -2.30 | 1.84E-02 | 34.4 | 5.1 |
| 52219007 | *zgc:92303* | -14.44 | -2.16 | 1.85E-02 | 257.5 | 41.7 |
| 83025077 | *zgc:123278* | -16.38 | -2.20 | 1.90E-02 | 68.5 | 10.7 |
| 189011581 | *hmox1* | -12.57 | -2.13 | 1.91E-02 | 931.8 | 154.0 |
| 146134892 | *crb3b* | -16.96 | -2.20 | 2.02E-02 | 45.6 | 7.2 |
| 189536214 | *si:dkeyp-27c8.2* | -17.75 | -2.30 | 2.07E-02 | 27.3 | 4.0 |
| 47086054 | *nr2f6b* | -19.30 | -2.53 | 2.26E-02 | 10.1 | 1.3 |
| 188536033 | *zgc:175140* | -19.32 | -2.49 | 2.50E-02 | 9.8 | 1.3 |
| 147905865 | *cyp24a1* | -12.40 | -2.01 | 2.59E-02 | 1010.7 | 180.8 |
| 189530840 | *LOC794786* | -18.34 | -2.23 | 2.61E-02 | 17.8 | 2.7 |
| 136256026 | *pygb* | -17.67 | -2.17 | 2.74E-02 | 27.6 | 4.4 |
| 47271548 | *junb* | -16.59 | -2.08 | 2.75E-02 | 56.8 | 9.7 |
| 66773047 | *tnfb* | -19.56 | -2.54 | 2.76E-02 | 8.5 | 1.1 |
| 47085980 | *nudcd3* | -18.82 | -2.32 | 2.78E-02 | 13.1 | 1.9 |
| 292621457 | *LOC100006782* | -12.73 | -1.99 | 2.79E-02 | 797.4 | 145.4 |
| 113951770 | *zgc:152809* | -10.71 | -1.96 | 2.95E-02 | 3213.3 | 596.0 |
| 66773152 | *zgc:110447* | -17.04 | -2.05 | 3.08E-02 | 41.0 | 7.2 |
| 47086190 | *otud3* | -19.59 | -2.49 | 3.11E-02 | 8.2 | 1.1 |
| 109254775 | *homez* | -15.18 | -1.96 | 3.13E-02 | 144.5 | 26.8 |
| 292627235 | *wu:fc21h01* | -18.87 | -2.23 | 3.26E-02 | 12.3 | 1.9 |
| 40363532 | *sgk1* | -14.91 | -1.93 | 3.37E-02 | 172.6 | 32.7 |
| 47086950 | *gng12a* | -15.61 | -1.94 | 3.41E-02 | 106.5 | 20.0 |
| 158517977 | *zgc:173927* | -18.61 | -2.17 | 3.41E-02 | 14.5 | 2.3 |
| 47087010 | *cog2* | -16.17 | -1.96 | 3.42E-02 | 72.6 | 13.5 |
| 224496031 | *LOC100148091* | -18.25 | -2.18 | 3.42E-02 | 18.6 | 2.9 |
| 148277606 | *si:ch211-149p10.2* | -17.92 | -2.13 | 3.42E-02 | 22.9 | 3.8 |
| 162138997 | *LOC564868* | -15.72 | -1.94 | 3.44E-02 | 98.9 | 18.5 |
| 50344805 | *fabp6* | -35.13 | -29.77 | 3.55E-02 | 2.2 | 0.0 |
| 160333656 | *acp6* | -17.80 | -2.06 | 3.80E-02 | 24.3 | 4.2 |
| 57768820 | *hsd17b14* | -15.76 | -1.90 | 3.80E-02 | 95.0 | 18.3 |
| 183583549 | *hsd11b3a* | -17.21 | -1.97 | 3.80E-02 | 35.5 | 6.5 |
| 118150623 | *fam46ba* | -18.27 | -2.14 | 3.82E-02 | 18.0 | 2.9 |
| 292620655 | *LOC565309* | -16.31 | -1.92 | 3.83E-02 | 65.3 | 12.4 |
| 225579105 | *glulb* | -18.10 | -2.09 | 3.88E-02 | 19.9 | 3.4 |
| 300934852 | *gck* | -15.90 | -1.90 | 3.89E-02 | 86.3 | 16.6 |
| 50540373 | *serpinb1* | -15.20 | -1.88 | 3.89E-02 | 138.5 | 27.2 |
| 47087320 | *gtpbp1* | -17.05 | -1.94 | 3.96E-02 | 39.3 | 7.4 |
| 117606282 | *irg1l* | -18.42 | -2.06 | 3.99E-02 | 15.8 | 2.7 |
| 45387810 | *fgf13b* | -18.78 | -2.11 | 4.04E-02 | 12.6 | 2.1 |
| 292611118 | *LOC100329302* | -8.05 | -1.83 | 4.17E-02 | 19307.8 | 3931.4 |
| 94536628 | *zgc:136439* | -18.65 | -2.09 | 4.22E-02 | 13.7 | 2.3 |
| 62955678 | *zgc:110152* | -15.63 | -1.85 | 4.29E-02 | 101.9 | 20.4 |
| 70887584 | *zgc:114058* | -19.08 | -2.15 | 4.34E-02 | 10.4 | 1.7 |
| 303227978 | *cltcb* | -16.71 | -1.90 | 4.35E-02 | 49.2 | 9.5 |
| 41055659 | *zgc:56112* | -18.79 | -2.07 | 4.37E-02 | 12.3 | 2.1 |
| 65301460 | *gadd45bb* | -19.66 | -2.34 | 4.51E-02 | 7.4 | 1.1 |
| 292617385 | *LOC555303* | -12.90 | -1.78 | 4.76E-02 | 658.7 | 138.6 |
| 292630196 | *LOC100334542* | -15.83 | -1.80 | 4.94E-02 | 87.4 | 18.1 |
| 41054763 | *sec31a* | -18.95 | -2.06 | 4.96E-02 | 10.9 | 1.9 |

| **G. KT11 up-regulated genes in male livers** | | | | | | |
| --- | --- | --- | --- | --- | --- | --- |
| GI_ID | Gene | logConc | logFC | P.Value | M_DMSO | M_KT11 |
| 123706334 | *zgc:158580* | -10.03 | 2.18 | 1.61E-02 | 1216.7 | 6145.8 |
| 169790921 | *dio2* | -12.28 | 2.67 | 3.86E-03 | 216.3 | 1534.5 |
| 158517993 | *cpb1* | -11.83 | 1.77 | 4.86E-02 | 404.5 | 1529.5 |
| 66773123 | *ela3l* | -13.00 | 2.25 | 1.34E-02 | 152.4 | 808.1 |
| 40548301 | *cpa5* | -12.84 | 1.91 | 3.45E-02 | 191.4 | 797.3 |
| 56799380 | *slc13a2* | -13.26 | 2.05 | 2.39E-02 | 136.0 | 625.2 |
| 56693266 | *sat2* | -14.27 | 3.50 | 2.83E-04 | 41.0 | 514.8 |
| 292611867 | *LOC100151267* | -13.83 | 2.42 | 8.38E-03 | 80.6 | 480.3 |
| 130488292 | *si:ch211-240l19.8* | -13.56 | 1.84 | 4.16E-02 | 119.3 | 473.4 |
| 55742560 | *chia.2* | -14.35 | 2.97 | 1.60E-03 | 46.4 | 405.1 |
| 110626116 | *si:ch211-240l19.5* | -14.23 | 2.40 | 9.10E-03 | 61.4 | 360.7 |
| 239582728 | *grn2* | -14.27 | 1.87 | 3.85E-02 | 72.1 | 293.7 |
| 292627402 | *LOC100330524* | -14.39 | 1.97 | 3.01E-02 | 64.2 | 279.6 |
| 292609495 | *si:dkeyp-75b4.10* | -15.04 | 2.78 | 3.12E-03 | 30.9 | 235.9 |
| 47086598 | *hsd11b2* | -14.80 | 2.22 | 1.56E-02 | 44.2 | 229.6 |
| 130487207 | *si:ch211-240l19.6* | -14.71 | 2.01 | 2.74E-02 | 50.5 | 226.3 |
| 292625551 | *LOC795071* | -14.68 | 1.91 | 3.50E-02 | 53.3 | 222.7 |
| 120586959 | *mhc1uda* | -14.71 | 1.92 | 3.48E-02 | 52.2 | 219.4 |
| 51226292 | *zgc:92137* | -16.21 | 3.91 | 1.03E-04 | 9.3 | 154.7 |
| 256418968 | *icn* | -15.15 | 1.78 | 4.91E-02 | 40.4 | 154.7 |
| 55742600 | *zgc:92590* | -15.58 | 2.11 | 2.18E-02 | 26.8 | 128.4 |
| 33636712 | *sult1st1* | -15.70 | 2.08 | 2.39E-02 | 24.9 | 116.6 |
| 41393162 | *mmp13a* | -15.86 | 2.28 | 1.42E-02 | 20.8 | 112.0 |
| 292610233 | *LOC555374* | -15.89 | 2.26 | 1.50E-02 | 20.5 | 109.4 |
| 41054372 | *calrl2* | -15.85 | 2.05 | 2.65E-02 | 22.7 | 104.5 |
| 292618123 | *LOC100329637* | -16.02 | 2.23 | 1.64E-02 | 18.8 | 98.2 |
| 292623984 | *zgc:162095* | -16.93 | 4.00 | 9.16E-05 | 5.5 | 96.9 |
| 292621639 | *LOC565781* | -16.61 | 3.27 | 9.18E-04 | 8.7 | 93.6 |
| 41055463 | *coro1a* | -16.33 | 2.66 | 5.12E-03 | 13.1 | 92.3 |
| 117606168 | *zgc:153073* | -15.97 | 1.84 | 4.58E-02 | 22.4 | 89.0 |
| 66472739 | *cpa1* | -16.16 | 2.18 | 1.87E-02 | 17.5 | 88.0 |
| 50539833 | *zgc:92061* | -16.07 | 1.89 | 4.04E-02 | 20.5 | 84.4 |
| 55742481 | *timp2b* | -16.45 | 2.56 | 7.04E-03 | 12.6 | 82.1 |
| 50540263 | *sult1st6* | -16.32 | 2.24 | 1.69E-02 | 15.3 | 80.5 |
| 139948508 | *zgc:158446* | -16.80 | 3.18 | 1.27E-03 | 7.9 | 79.5 |
| 47086594 | *mknk2a* | -16.41 | 2.27 | 1.58E-02 | 14.2 | 76.2 |
| 41282139 | *cebpa* | -16.33 | 2.02 | 3.06E-02 | 16.4 | 73.9 |
| 40353234 | *aif1l* | -16.41 | 2.06 | 2.74E-02 | 15.3 | 71.0 |
| 55741883 | *abcc4* | -16.32 | 1.85 | 4.42E-02 | 17.5 | 70.0 |
| 50344837 | *cyp2aa4* | -16.49 | 2.11 | 2.47E-02 | 14.2 | 68.0 |
| 292612298 | *LOC100331950* | -17.12 | 3.21 | 1.24E-03 | 6.3 | 64.4 |
| 51556246 | *zgc:77395* | -16.67 | 2.18 | 2.04E-02 | 12.3 | 61.8 |
| 70887622 | *zgc:112368* | -16.66 | 1.94 | 3.77E-02 | 13.4 | 57.2 |
| 41054646 | *slc2a12* | -16.74 | 1.91 | 4.08E-02 | 12.8 | 53.5 |
| 113674586 | *pkp3* | -16.71 | 1.85 | 4.72E-02 | 13.4 | 53.5 |
| 50582983 | *cxcl12a* | -16.83 | 2.04 | 2.96E-02 | 11.5 | 52.6 |
| 53749654 | *psmb9a* | -16.97 | 2.22 | 2.07E-02 | 9.8 | 50.9 |
| 52218895 | *fkbp3* | -16.92 | 1.86 | 4.61E-02 | 11.5 | 46.3 |
| 150378484 | *LOC791723* | -17.91 | 3.75 | 4.42E-04 | 3.0 | 45.0 |
| 56693260 | *gcga* | -17.62 | 3.08 | 2.11E-03 | 4.6 | 43.7 |
| 47271424 | *sult1st2* | -18.20 | 4.09 | 2.01E-04 | 2.2 | 41.4 |
| 115495218 | *itln1* | -17.97 | 3.39 | 9.41E-04 | 3.3 | 38.1 |
| 160425212 | *si:dkey-239i20.2* | -17.45 | 2.30 | 1.76E-02 | 6.8 | 37.5 |
| 292627816 | *LOC792236* | -18.81 | 4.87 | 4.46E-05 | 1.1 | 35.5 |
| 55742387 | *si:busm1-266f07.1* | -17.97 | 3.16 | 1.92E-03 | 3.6 | 35.2 |
| 115496863 | *ptk6b* | -17.85 | 2.63 | 8.13E-03 | 4.6 | 31.9 |
| 157743301 | *anxa2b* | -17.69 | 2.32 | 1.77E-02 | 5.7 | 31.9 |
| 47575846 | *mao* | -17.60 | 2.13 | 2.84E-02 | 6.6 | 31.9 |
| 18858428 | *cldn15la* | -17.97 | 2.75 | 6.01E-03 | 4.1 | 30.6 |
| 47550906 | *fabp7b* | -17.76 | 2.33 | 1.74E-02 | 5.5 | 30.6 |
| 41054919 | *pdia2* | -18.80 | 4.25 | 2.46E-04 | 1.4 | 28.9 |
| 50539793 | *cd36* | -17.74 | 1.97 | 4.40E-02 | 6.3 | 27.3 |
| 47085748 | *itpk1* | -18.61 | 3.66 | 9.95E-04 | 1.9 | 26.9 |
| 292616117 | *LOC567192* | -18.17 | 2.75 | 6.45E-03 | 3.6 | 26.6 |
| 62955330 | *zgc:112015* | -17.97 | 2.21 | 2.51E-02 | 4.9 | 25.3 |
| 48762656 | *eno1* | -18.32 | 2.68 | 8.15E-03 | 3.3 | 23.3 |
| 116875802 | *zgc:152682* | -18.41 | 2.76 | 9.12E-03 | 3.0 | 22.7 |
| 292625156 | *LOC566752* | -18.56 | 3.03 | 5.03E-03 | 2.5 | 22.3 |
| 189519947 | *LOC565984* | -19.39 | 4.55 | 2.37E-04 | 0.8 | 21.4 |
| 300795235 | *prkar1aa* | -18.82 | 3.24 | 3.77E-03 | 1.9 | 20.0 |
| 123707182 | *bckdhb* | -18.24 | 2.02 | 4.27E-02 | 4.4 | 19.7 |
| 269847758 | *slco2b1* | -18.38 | 2.11 | 3.53E-02 | 3.8 | 18.4 |
| 47085868 | *mmp9* | -18.58 | 2.41 | 2.43E-02 | 3.0 | 17.7 |
| 169259805 | *LOC100141478* | -18.52 | 2.28 | 2.43E-02 | 3.3 | 17.7 |
| 292610866 | *LOC567822* | -18.46 | 2.17 | 3.15E-02 | 3.6 | 17.7 |
| 45387526 | *smyd1a* | -19.55 | 4.23 | 6.99E-04 | 0.8 | 17.1 |
| 236464255 | *mxc* | -18.55 | 2.23 | 2.77E-02 | 3.3 | 17.1 |
| 41152339 | *lsm4* | -18.65 | 2.27 | 3.41E-02 | 3.0 | 16.1 |
| 70912383 | *tmem37* | -19.91 | 4.67 | 4.41E-04 | 0.5 | 15.4 |
| 292630419 | *gcgb* | -19.01 | 2.86 | 1.06E-02 | 1.9 | 15.4 |
| 41054813 | *ero1l* | -18.80 | 2.25 | 3.74E-02 | 2.7 | 14.5 |
| 47086680 | *hmga2* | -19.48 | 3.54 | 3.23E-03 | 1.1 | 14.1 |
| 292623257 | *LOC556873* | -18.89 | 2.37 | 3.01E-02 | 2.5 | 14.1 |
| 50345091 | *cpa4* | -19.09 | 2.70 | 1.61E-02 | 1.9 | 13.8 |
| 157311768 | *si:ch211-137i24.5* | -18.85 | 2.15 | 4.73E-02 | 2.7 | 13.5 |
| 139948744 | *ghrl* | -20.53 | 5.44 | 2.80E-04 | 0.3 | 13.1 |
| 292617823 | *si:dkey-79p17.2* | -20.03 | 4.44 | 8.91E-04 | 0.5 | 13.1 |
| 292622287 | *LOC563795* | -19.24 | 2.85 | 1.26E-02 | 1.6 | 13.1 |
| 188536021 | *zgc:175175* | -18.94 | 2.27 | 3.84E-02 | 2.5 | 13.1 |
| 295834926 | *nr0b2a* | -19.05 | 2.40 | 3.03E-02 | 2.2 | 12.8 |
| 50344805 | *fabp6* | -19.07 | 2.36 | 3.62E-02 | 2.2 | 12.5 |
| 55925250 | *zgc:103585* | -18.98 | 2.19 | 4.96E-02 | 2.5 | 12.5 |
| 55925188 | *zgc:101897* | -19.29 | 2.74 | 1.85E-02 | 1.6 | 12.2 |
| 292627814 | *LOC798251* | -20.65 | 5.20 | 6.43E-04 | 0.3 | 11.2 |
| 160333639 | *snx10b* | -19.35 | 2.62 | 2.50E-02 | 1.6 | 11.2 |
| 115497275 | *zgc:153118* | -19.24 | 2.39 | 3.71E-02 | 1.9 | 11.2 |
| 150378482 | *hspb11* | -19.24 | 2.39 | 3.71E-02 | 1.9 | 11.2 |
| 113931645 | *zgc:153896* | -19.90 | 3.53 | 5.86E-03 | 0.8 | 10.5 |
| 62955788 | *zgc:112992* | -19.53 | 2.79 | 1.96E-02 | 1.4 | 10.5 |
| 292619336 | *wu:fb81h03* | -19.29 | 2.31 | 4.54E-02 | 1.9 | 10.5 |
| 292616584 | *LOC793037* | -20.76 | 4.97 | 1.27E-03 | 0.3 | 9.5 |
| 41054500 | *zbtb16a* | -20.24 | 2.85 | 3.15E-02 | 0.8 | 6.6 |
| 283436089 | *sc:d0261* | -21.07 | 4.36 | 6.60E-03 | 0.3 | 6.2 |
| 45387754 | *zgc:77158* | -20.31 | 2.70 | 4.38E-02 | 0.8 | 5.9 |
| 47824884 | *mid1ip1* | -20.79 | 2.92 | 4.68E-02 | 0.5 | 4.6 |

| **H. KT11 down-regulated genes in male livers** | | | | | | |
| --- | --- | --- | --- | --- | --- | --- |
| GI_ID | Gene | logConc | logFC | P.Value | M_DMSO | M_KT11 |
| 166795886 | *vtg1* | -9.87 | -5.01 | 1.06E-06 | 16449.5 | 567.7 |
| 160420305 | *vtg4* | -10.33 | -3.93 | 5.61E-05 | 8281.4 | 602.5 |
| 221307487 | *si:ch1073-126c3.2* | -10.66 | -4.23 | 1.90E-05 | 7303.7 | 431.7 |
| 68448529 | *vtg5* | -11.32 | -3.50 | 2.61E-04 | 3570.2 | 351.8 |
| 229335594 | *rpl39* | -11.97 | -2.89 | 1.99E-03 | 1844.5 | 277.3 |
| 50539815 | *zgc:92066* | -13.00 | -4.87 | 1.90E-06 | 1800.0 | 68.3 |
| 41055254 | *rps21* | -12.04 | -1.87 | 3.73E-02 | 1238.2 | 375.8 |
| 113678457 | *vtg2* | -13.56 | -4.23 | 2.08E-05 | 976.9 | 57.8 |
| 41056076 | *rps3* | -12.93 | -2.09 | 2.13E-02 | 716.3 | 187.3 |
| 115529346 | *rps16* | -12.93 | -1.85 | 3.96E-02 | 660.6 | 203.3 |
| 48597011 | *rpl23a* | -13.74 | -3.35 | 4.64E-04 | 635.0 | 69.3 |
| 115497491 | *pmt* | -13.34 | -2.41 | 8.82E-03 | 604.6 | 126.8 |
| 50540201 | *gc* | -13.61 | -2.31 | 1.17E-02 | 485.3 | 109.1 |
| 154147743 | *rnasel2* | -13.62 | -2.22 | 1.49E-02 | 465.6 | 110.7 |
| 41053334 | *rps26* | -15.36 | -3.16 | 9.80E-04 | 192.5 | 24.0 |
| 157073892 | *atf4b2* | -15.34 | -2.77 | 3.33E-03 | 171.8 | 27.9 |
| 33504546 | *serpind1* | -15.15 | -2.03 | 2.65E-02 | 150.8 | 41.1 |
| 41152011 | *lrpap1* | -15.32 | -2.20 | 1.69E-02 | 142.6 | 34.5 |
| 189536130 | *si:ch211-66i15.5* | -16.37 | -4.24 | 3.31E-05 | 140.1 | 8.2 |
| 160333704 | *vtg2* | -16.65 | -3.79 | 1.68E-04 | 98.3 | 7.9 |
| 187608538 | *si:ch211-235e18.3* | -15.93 | -2.15 | 2.05E-02 | 92.0 | 23.0 |
| 50539951 | *cldn15lb* | -16.09 | -2.47 | 8.60E-03 | 91.5 | 18.4 |
| 125845146 | *nr0b2b* | -16.79 | -3.76 | 1.98E-04 | 88.2 | 7.2 |
| 62955574 | *psmb4* | -16.50 | -3.17 | 1.12E-03 | 87.7 | 10.8 |
| 300934852 | *gck* | -16.56 | -3.24 | 9.28E-04 | 86.3 | 10.2 |
| 115529362 | *zgc:153027* | -17.22 | -4.21 | 6.37E-05 | 76.7 | 4.6 |
| 50539803 | *tm4sf5* | -16.10 | -1.82 | 4.86E-02 | 72.9 | 23.0 |
| 189529978 | *si:dkey-97i18.2* | -16.37 | -2.29 | 1.47E-02 | 71.0 | 16.1 |
| 41055467 | *gpr39* | -16.39 | -1.87 | 4.39E-02 | 60.4 | 18.4 |
| 157841229 | *cyp2aa7* | -17.34 | -3.58 | 4.86E-04 | 56.5 | 5.3 |
| 61806623 | *c6ast3* | -16.77 | -2.08 | 2.82E-02 | 50.0 | 13.1 |
| 55742572 | *slc25a33* | -16.85 | -1.85 | 4.98E-02 | 43.7 | 13.5 |
| 189521420 | *intl2* | -17.65 | -3.35 | 1.19E-03 | 42.1 | 4.6 |
| 45598387 | *zgc:77655* | -17.03 | -2.04 | 3.08E-02 | 41.2 | 11.2 |
| 46358343 | *idh3a* | -17.08 | -1.94 | 3.90E-02 | 38.5 | 11.2 |
| 40789251 | *rpl36a* | -17.38 | -2.45 | 1.20E-02 | 37.1 | 7.6 |
| 287327509 | *sepx1b* | -17.20 | -1.86 | 4.87E-02 | 34.4 | 10.5 |
| 18858810 | *hhex* | -18.73 | -4.80 | 3.74E-05 | 33.0 | 1.3 |
| 303227888 | *vtg6* | -17.96 | -3.17 | 2.37E-03 | 32.0 | 3.9 |
| 47085886 | *marcksl1* | -17.50 | -2.10 | 3.03E-02 | 30.3 | 7.9 |
| 57526004 | *pltp* | -18.22 | -3.48 | 8.82E-04 | 29.8 | 3.0 |
| 62955548 | *zgc:110349* | -18.61 | -3.89 | 4.16E-04 | 26.2 | 2.0 |
| 134133299 | *entpd3* | -19.91 | -6.44 | 6.49E-06 | 25.7 | 0.3 |
| 292618008 | *LOC100334232* | -18.22 | -2.90 | 5.85E-03 | 24.3 | 3.6 |
| 50344853 | *slc25a11* | -17.93 | -2.07 | 3.75E-02 | 22.4 | 5.9 |
| 56797743 | *srsf6b* | -18.25 | -2.60 | 1.27E-02 | 21.6 | 3.9 |
| 47085910 | *lsm12* | -19.26 | -4.59 | 1.48E-04 | 21.3 | 1.0 |
| 292624225 | *LOC100151336* | -18.14 | -2.36 | 2.13E-02 | 21.3 | 4.6 |
| 115497451 | *zgc:153138* | -33.52 | -33.00 | 3.42E-06 | 20.5 | 0.0 |
| 47085734 | *dip2ba* | -18.09 | -2.10 | 3.88E-02 | 20.2 | 5.3 |
| 292618717 | *vtg3* | -18.69 | -3.27 | 2.39E-03 | 19.9 | 2.3 |
| 225579105 | *glulb* | -18.19 | -2.27 | 2.76E-02 | 19.9 | 4.6 |
| 121583941 | *zgc:153790* | -18.45 | -2.71 | 8.45E-03 | 19.4 | 3.3 |
| 54400451 | *zgc:103599* | -19.63 | -5.01 | 9.94E-05 | 19.1 | 0.7 |
| 121582451 | *znf395a* | -19.00 | -3.63 | 1.28E-03 | 18.3 | 1.6 |
| 292610905 | *si:dkeyp-55c8.3* | -19.04 | -3.54 | 1.58E-03 | 17.2 | 1.6 |
| 292626279 | *wu:fb17f05* | -19.43 | -4.23 | 4.64E-04 | 16.7 | 1.0 |
| 292612076 | *LOC100334641* | -18.65 | -2.32 | 2.40E-02 | 14.7 | 3.3 |
| 50539751 | *zgc:92181* | -18.67 | -2.29 | 2.58E-02 | 14.5 | 3.3 |
| 292624905 | *si:ch211-153j24.6* | -18.69 | -2.24 | 2.99E-02 | 13.9 | 3.3 |
| 156713466 | *vtg7* | -19.37 | -3.53 | 2.45E-03 | 13.7 | 1.3 |
| 292625448 | *LOC564720* | -18.99 | -2.66 | 1.36E-02 | 13.1 | 2.3 |
| 62955242 | *zgc:110234* | -19.62 | -3.86 | 1.59E-03 | 12.8 | 1.0 |
| 292627235 | *wu:fc21h01* | -19.15 | -2.79 | 1.15E-02 | 12.3 | 2.0 |
| 62955240 | *naa40* | -19.18 | -2.73 | 1.38E-02 | 11.7 | 2.0 |
| 292627179 | *nr1d4a* | -19.99 | -4.28 | 1.11E-03 | 11.5 | 0.7 |
| 292624161 | *LOC100001036* | -19.49 | -3.28 | 5.29E-03 | 11.5 | 1.3 |
| 300796163 | *ttc27* | -19.07 | -2.13 | 4.34E-02 | 10.4 | 2.6 |
| 146134959 | *cyp2aa1* | -19.09 | -2.10 | 4.76E-02 | 10.1 | 2.6 |
| 292615004 | *raver2* | -20.13 | -4.01 | 2.24E-03 | 9.6 | 0.7 |
| 292628115 | *LOC100332146* | -20.13 | -4.01 | 2.24E-03 | 9.6 | 0.7 |
| 54400559 | *trappc6b* | -19.63 | -3.01 | 1.01E-02 | 9.6 | 1.3 |
| 292611679 | *LOC100330261* | -20.93 | -4.41 | 4.56E-03 | 6.3 | 0.3 |
| 18859120 | *nots* | -34.90 | -30.23 | 1.32E-02 | 3.0 | 0.0 |
| 18859574 | *zp3b* | -34.97 | -30.09 | 1.80E-02 | 2.7 | 0.0 |
| 167963457 | *zgc:174710* | -35.05 | -29.94 | 2.50E-02 | 2.5 | 0.0 |
