# Supplementary material for: Transcriptomic Analyses of Sexual Dimorphism of the Zebrafish Liver and the Effect of Sex Hormones
Source: PLoS One. 2013 Jan 17;8(1):e53562. doi: 10.1371/journal.pone.0053562 (PMC3547925; doi:10.1371/journal.pone.0053562)
Supplement: Table S5 — Gene ontology enrichment analysis of E2- and KT11-induced transcriptome changes in the male liver. (DOCX) [file pone.0053562.s006.docx]

**Table S5. Gene ontology enrichment analysis of E2- and KT11-induced transcriptome changes in the male liver**

| **A. GO enrichment analysis for E2 induced transcriptomic changes in the male liver** | | | | | | |
| --- | --- | --- | --- | --- | --- | --- |
|  | | Term | Count | % | Fold Enrich | PValue |
| Up | BP | GO:0055114~oxidation reduction | 22 | 12.94 | 3.28 | 1.80E-06 |
|  |  | GO:0009725~response to hormone stimulus | 4 | 2.35 | 18.76 | 1.10E-03 |
|  |  | GO:0043627~response to estrogen stimulus | 3 | 1.76 | 45.02 | 1.68E-03 |
|  |  | GO:0032355~response to estradiol stimulus | 3 | 1.76 | 45.02 | 1.68E-03 |
|  |  | GO:0009719~response to endogenous stimulus | 4 | 2.35 | 15.80 | 1.85E-03 |
|  |  | GO:0006417~regulation of translation | 5 | 2.94 | 9.15 | 1.99E-03 |
|  |  | GO:0010033~response to organic substance | 5 | 2.94 | 7.82 | 3.56E-03 |
|  |  | GO:0048545~response to steroid hormone stimulus | 3 | 1.76 | 28.14 | 4.58E-03 |
|  |  | GO:0032268~regulation of cellular protein metabolic process | 5 | 2.94 | 6.82 | 5.81E-03 |
|  |  | GO:0010817~regulation of hormone levels | 3 | 1.76 | 14.07 | 1.83E-02 |
|  |  | GO:0042445~hormone metabolic process | 3 | 1.76 | 14.07 | 1.83E-02 |
|  |  | GO:0006869~lipid transport | 4 | 2.35 | 6.67 | 2.12E-02 |
|  |  | GO:0010876~lipid localization | 4 | 2.35 | 6.53 | 2.25E-02 |
|  | MF | GO:0004181~metallocarboxypeptidase activity | 5 | 2.94 | 20.69 | 7.87E-05 |
|  |  | GO:0004180~carboxypeptidase activity | 5 | 2.94 | 16.19 | 2.16E-04 |
|  |  | GO:0008235~metalloexopeptidase activity | 5 | 2.94 | 14.90 | 3.03E-04 |
|  |  | GO:0003735~structural constituent of ribosome | 8 | 4.71 | 4.20 | 2.78E-03 |
|  |  | GO:0008238~exopeptidase activity | 5 | 2.94 | 8.10 | 3.15E-03 |
|  |  | GO:0020037~heme binding | 7 | 4.12 | 4.35 | 5.19E-03 |
|  |  | GO:0005506~iron ion binding | 10 | 5.88 | 2.96 | 6.29E-03 |
|  |  | GO:0046906~tetrapyrrole binding | 7 | 4.12 | 4.14 | 6.57E-03 |
|  |  | GO:0016702~oxidoreductase activity, acting on single donors with incorporation of molecular oxygen, incorporation of two atoms of oxygen | 4 | 2.35 | 9.03 | 9.36E-03 |
|  |  | GO:0016701~oxidoreductase activity, acting on single donors with incorporation of molecular oxygen | 4 | 2.35 | 8.76 | 1.02E-02 |
|  |  | GO:0009055~electron carrier activity | 8 | 4.71 | 3.24 | 1.12E-02 |
|  |  | GO:0004800~thyroxine 5'-deiodinase activity | 2 | 1.18 | 74.49 | 2.64E-02 |
|  |  | GO:0005319~lipid transporter activity | 3 | 1.76 | 10.64 | 3.13E-02 |
|  |  | GO:0004104~cholinesterase activity | 2 | 1.18 | 49.66 | 3.94E-02 |
|  | CC | GO:0005840~ribosome | 8 | 4.71 | 4.74 | 1.07E-03 |
|  |  | GO:0043228~non-membrane-bounded organelle | 14 | 8.24 | 2.09 | 8.90E-03 |
|  |  | GO:0043232~intracellular non-membrane-bounded organelle | 14 | 8.24 | 2.09 | 8.90E-03 |
|  |  | GO:0030529~ribonucleoprotein complex | 8 | 4.71 | 3.03 | 1.29E-02 |
| Down | BP | GO:0055114~oxidation reduction | 8 | 8.42 | 2.65 | 2.59E-02 |
|  | MF | GO:0020037~heme binding | 4 | 4.21 | 5.73 | 3.07E-02 |
|  |  | GO:0046906~tetrapyrrole binding | 4 | 4.21 | 5.45 | 3.48E-02 |

| **A. GO enrichment analysis for KT11 induced transcriptomic changes in the male liver** | | | | | | |
| --- | --- | --- | --- | --- | --- | --- |
|  | | Term | Count | % | Fold Enrich | PValue |
| Up | BP | GO:0006508~proteolysis | 12 | 14.81 | 3.50 | 3.61E-04 |
|  |  | GO:0018958~phenol metabolic process | 3 | 3.70 | 47.90 | 1.60E-03 |
|  |  | GO:0006584~catecholamine metabolic process | 3 | 3.70 | 47.90 | 1.60E-03 |
|  |  | GO:0009712~catechol metabolic process | 3 | 3.70 | 47.90 | 1.60E-03 |
|  |  | GO:0034311~diol metabolic process | 3 | 3.70 | 47.90 | 1.60E-03 |
|  |  | GO:0006955~immune response | 6 | 7.41 | 5.74 | 3.37E-03 |
|  |  | GO:0006576~biogenic amine metabolic process | 3 | 3.70 | 20.83 | 8.57E-03 |
|  |  | GO:0006575~cellular amino acid derivative metabolic process | 3 | 3.70 | 12.60 | 2.25E-02 |
|  |  | GO:0006805~xenobiotic metabolic process | 2 | 2.47 | 79.83 | 2.43E-02 |
|  |  | GO:0019882~antigen processing and presentation | 3 | 3.70 | 9.21 | 4.02E-02 |
|  | MF | GO:0070011~peptidase activity, acting on L-amino acid peptides | 12 | 14.81 | 4.26 | 7.30E-05 |
|  |  | GO:0008233~peptidase activity | 12 | 14.81 | 4.06 | 1.13E-04 |
|  |  | GO:0004181~metallocarboxypeptidase activity | 4 | 4.94 | 33.40 | 2.01E-04 |
|  |  | GO:0004180~carboxypeptidase activity | 4 | 4.94 | 26.14 | 4.26E-04 |
|  |  | GO:0008235~metalloexopeptidase activity | 4 | 4.94 | 24.05 | 5.48E-04 |
|  |  | GO:0008237~metallopeptidase activity | 6 | 7.41 | 7.33 | 1.19E-03 |
|  |  | GO:0004175~endopeptidase activity | 8 | 9.88 | 4.10 | 2.79E-03 |
|  |  | GO:0008238~exopeptidase activity | 4 | 4.94 | 13.07 | 3.29E-03 |
|  |  | GO:0004304~estrone sulfotransferase activity | 2 | 2.47 | 150.30 | 1.30E-02 |
|  |  | GO:0008289~lipid binding | 5 | 6.17 | 4.34 | 2.61E-02 |
|  |  | GO:0008146~sulfotransferase activity | 3 | 3.70 | 11.00 | 2.92E-02 |
|  |  | GO:0016782~transferase activity, transferring sulfur-containing groups | 3 | 3.70 | 9.59 | 3.76E-02 |
|  |  | GO:0004252~serine-type endopeptidase activity | 4 | 4.94 | 4.97 | 4.43E-02 |
|  | CC | GO:0005576~extracellular region | 9 | 11.11 | 3.32 | 2.84E-03 |
|  |  | GO:0042611~MHC protein complex | 3 | 3.70 | 10.41 | 3.07E-02 |
| Down | BP | GO:0006412~translation | 7 | 12.50 | 6.31 | 5.79E-04 |
|  |  | GO:0006869~lipid transport | 3 | 5.36 | 15.63 | 1.46E-02 |
|  |  | GO:0010876~lipid localization | 3 | 5.36 | 15.29 | 1.53E-02 |
|  |  | GO:0019882~antigen processing and presentation | 3 | 5.36 | 13.53 | 1.93E-02 |
|  |  | GO:0032355~response to estradiol stimulus | 2 | 3.57 | 93.80 | 2.05E-02 |
|  |  | GO:0043627~response to estrogen stimulus | 2 | 3.57 | 93.80 | 2.05E-02 |
|  |  | GO:0048545~response to steroid hormone stimulus | 2 | 3.57 | 58.63 | 3.26E-02 |
|  | MF | GO:0003735~structural constituent of ribosome | 7 | 12.50 | 10.37 | 4.28E-05 |
|  |  | GO:0005198~structural molecule activity | 8 | 14.29 | 5.28 | 5.68E-04 |
|  |  | GO:0005319~lipid transporter activity | 3 | 5.36 | 30.06 | 4.16E-03 |
|  | CC | GO:0005840~ribosome | 7 | 12.50 | 7.75 | 1.48E-04 |
|  |  | GO:0030529~ribonucleoprotein complex | 7 | 12.50 | 4.95 | 1.64E-03 |
|  |  | GO:0042611~MHC protein complex | 3 | 5.36 | 11.77 | 2.41E-02 |
